# Supplementary material for: Unaddressed non-energy use in the chemical industry can undermine fossil fuels phase-out
Source: Nat Commun. 2024 Sep 14;15:8050. doi: 10.1038/s41467-024-52434-y (PMC11401860; doi:10.1038/s41467-024-52434-y)
Supplement: Supplementary file 1 — Supplementary Information [file 41467_2024_52434_MOESM1_ESM.pdf]

Supplementary Information for:

Unaddressed non-energy use in the chemical industry can undermine fossil fuels phase-out

Marianne Zanon-Zotin[1,2], Luiz Bernardo Baptista[1,3], Rebecca Draeger[1], Pedro R. R. Rochedo[4*], Alexandre Szklo[1], Roberto Schaeffer[1]

[1] Centre for Energy and Environmental Economics (Cenergia), Energy Planning Program (PPE), COPPE, Universidade Federal do Rio de Janeiro, Brazil

[2] Copernicus Institute of Sustainable Development, Utrecht University, Utrecht, The Netherlands

[3] PBL Netherlands Environmental Assessment Agency, The Hague, The Netherlands

[4] Research and Innovation Center on CO2 and Hydrogen (RICH Center) and Management Science and Engineering Department, Khalifa University, Abu Dhabi, United Arab Emirates

* Corresponding author, pedro.rochedo@ku.ac.ae

These Supplementary Information includes:
Supplementary Method 1 – 6
Supplementary Table 1-4
Supplementary Figure 1-17
Supplementary References

Supplementary Method 1. Sources of carbon feedstock

COFFEE represents fossil-, bio- and CCU-based feedstocks as options for producing primary chemicals.

Fossil-based feedstock: Primarily includes natural gas (i.e., methane and ethane), oil (i.e., LPG, naphtha and heavy oil), and coal. Natural gas can be produced in fields associated with oil or in dedicated fields. Ethane and methane can be recovered from natural gas and used for steam cracking and steam methane reforming units, respectively. LPG, naphtha and heavy oil are outputs of the petroleum refining sector, which is described in ref.[1]. LPG is an input to propane dehydrogenation process, naphtha can be used in steam cracking and catalytic reforming units whereas heavy oil is used for hydrogen to ammonia production via partial oil oxidation. Coal is produced in different qualities in the resource module (bituminous, subbituminous, and lignite) but only bituminous coal is allowed in coal gasification processes.

Bio-based feedstock: Primarily includes solid and liquid biofuel produced from planted forests and energy crops. Solid biofuel is obtained from woody (eucalyptus) and grassy sources as well as agriculture residues, including sugarcane bagasse. Eucalyptus can be used for biomass gasification processes to produce hydrogen (for energy or ammonia production), methanol, or can be used in Fischer-Tropsch Biomass-to-Liquids (FT-BtL) to produce advanced liquid biofuels. Sugarcane bagasse can be used for 2G bioethanol production.

Liquid biofuels included as feedstocks for the chemical sector are bioethanol (via fermentation/distillation from sugarcane, maize, wheat, sugar beet, and agricultural residues), and bio-naphtha, and bio-LPG as co-products of hydrotreated vegetable oils (HVO) or hydroprocessed esters and fatty acids (HEFA) production from soybean oil, maize oil, or animal fat. HVO and HEFA are advanced biofuels alternatives to be used in diesel engines as green diesel or jet fuel (or sustainable aviation fuel – SAF), and might co-produce bio-naphtha and bio-LPG to be used in other sectors, including as feedstocks.

Biomethane could be a promising alternative feedstock. However, its supply chain is not yet developed in COFFEE and it remains as a topic to be further explored in future work.

CCU-based feedstock: CO_2 as a feedstock is sourced from a "CO_2 pool", which is composed by CO_2 captured from: 1) <u>fossil- or bio-based concentrated sources</u> such as power plants, cement kilns, blast furnaces, and methanol/ammonia plants (via gasification/partial oxidation, but not electrolysis). Both energy and process emissions can be captured from concentrated

sources, and costs for CO_2 transportation are considered; 2) diluted CO_2 in the atmosphere via Direct Air Capture (DAC) based on absorption in sodium hydroxide solution, based on refs,[2,3]. Captured CO_2 feeds into a regional “CO_2 pool” in the model, where the model decides whether it is geologically stored or used for CCU.

Supplementary Method 2. Technology representation

In this study, we categorize ammonia, methanol and high-value chemicals (i.e., ethylene, propylene, butadiene and BTX) as primary chemicals. They are the building blocks in multiple downstream processes that convert them into more complex chemicals for a multitude of material services. While capturing the use of primary and final energy sources for combustion in IAMs is critical to evaluate the climate-related challenges associated with replacing the primary energy source in the current global economy, it is equally important to understand their non-energy uses. This detailed perspective helps us to identify the broader implications of replacing fossil fuels, given their ubiquitous role in society as sources of both energy and materials.

The following sections provide details on technoeconomic parameters and other relevant data of the primary chemicals technologies included in the COFFEE model. Supplementary Figure 1 presents the technologies included in the primary chemicals module and their respective products, as well as the interaction with the oil refining module. Supplementary Table 1, Supplementary Table *2*, and Supplementary Table 3 summarize the techno-economic parameters used in this study.

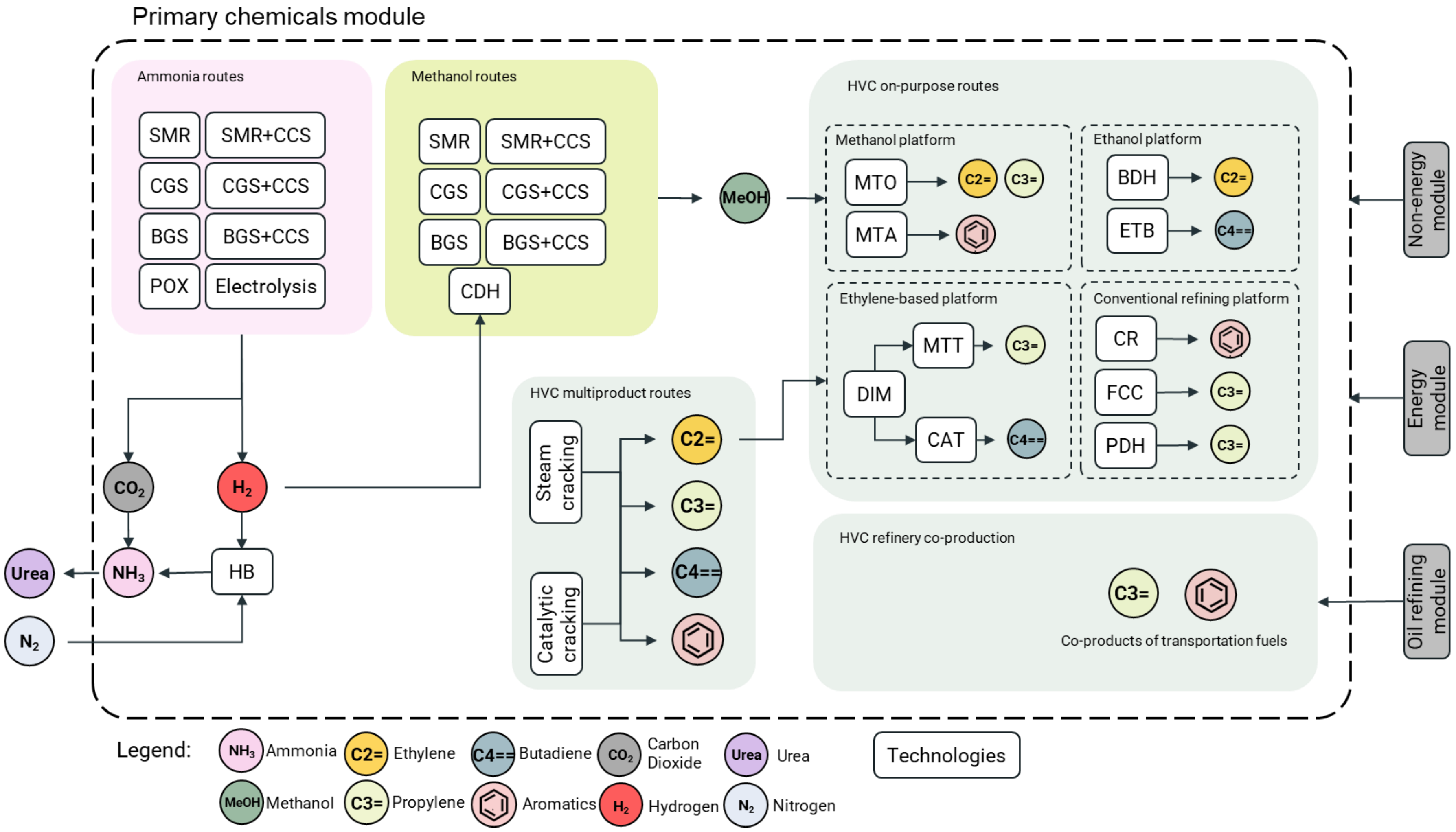

Supplementary Figure 1. Primary chemicals module represented in the COFFEE model.

*S*MR: Steam Methane Reforming; CGS: Coal Gasification; BGS: Biomass Gasification; POX: Partial Oxidation of Oil; HB: Haber Bosch synthesis; CDH: Carbon Dioxide Hydrogenation; DIM: Dimerization; CAT: Catadiene®; MTT: Metathesis; CR: Catalytic Reforming; FCC: Fluidized Catalytic Cracking; PDH: Propane Dehydrogenation; BDH: Bioethanol Dehydration; ETB: Ethanol to Butadiene; MTO: Methanol-to-Olefins; MTA: Methanol-to-Aromatics

High-value Chemicals

High Value Chemicals (HVCs) are basic chemicals widely used as building blocks for numerous chemical products. **Ethylene** (C_2H_4) is the primary raw material for the downstream petrochemical industry, and its main derivatives include (high-density, low-density, and linear low-density) polyethylene, polyethylene terephthalate (PET), ethylene oxide (intermediate for ethylene glycol), ethylene dichloride (precursor of PVC), and ethylbenzene (intermediate of polystyrene - PS), among others. Global ethylene production – approximately 190 $Mt.yr^{-1}$ – has been steadily growing over the past decades.

Propylene (C_3H_6) is the second basic chemical in importance, being the building block for several polymers and synthetic fibres such as polypropylene, propylene oxide, acrylonitrile, and others. Global propylene production reached around 85 Mt in 2015 [4].

Butadiene (C_4H_6) is a linear diolefin produced as a by-product of ethylene with a global production capacity around 11 $Mt.yr^{-1}$ [5,6]. It is mostly used for rubbers such as styrene-butadiene rubber (SBR) and polybutadiene (PB), which are essential for tires production, but also acrylonitrile-butadiene-styrene resins (ABS) and other rubbers.

BTX stands for **Benzene** (C_6H_6), **Toluene** (C_7H_8) and **Xylenes** (C_8H_{10}, which includes ortho, meta and para isomers) and global production is approximately of 95 $Mt.yr^{-1}$ [7]. While p-xylene is mostly used for terephthalic acid production (i.e., PET precursor), benzene applications are more diverse such as for ethylbenzene (for PS production), cumene and cyclohexane, chemicals widely used as solvents, paints, and intermediates. In this study, the terms *aromatics* and *BTX* are used interchangeably.

Thus, by including these products in an IAM, we aim to capture the role of downstream chemicals produced in large scale such as plastics, synthetic fibres and rubbers in future energy and carbon feedstock demand as well as carbon emissions. HVCs technologies are categorized as <u>Refinery co-production</u>, <u>Multiproduct</u> and <u>On-purpose</u> (i.e., facilities dedicated to produce a specific primary chemical, as opposed to steam cracking) to account for potential structural changes that the petrochemical sector may experience due to feedstock transition and climate stringent targets. Below, we describe each technology and present the modelling strategy to represent this sector as well as data sources for regional installed capacity in the base year. Techno-economic assumptions for all technologies are also summarized in Supplementary Table 1.

- Refinery co-production technologies (FCC-o and CR-o)

A significant share of the global propylene and BTX production is recovered from oil refineries, mainly in gasoline-producing refineries Fluidized Catalytic Cracking (FCC) and Catalytic Reforming (CR) units, respectively. While propylene is recovered diluted in the propane stream in the FCC unit, BTX is produced in Catalytic Reforming unit primarily as an octane rating booster for gasoline but also as a building block for polymers production.

Regional propylene and BTX recovery from FCC and RC were assumed based on country-specific data from the OGJ Worldwide Refining Survey [8]. While aromatics production is explicitly informed in the referred reference, propylene recovery yield in FCC units was assumed to be 5.0 wt% on fresh feed [9].

In the COFFEE model, three refinery schemes (Cracking and Coking, Topping and Hydroskimming, and Hycon), seven oil qualities (Light, Medium Sweet, Medium Sour, Heavy, Extra Heavy, Bitumen, and Kerogen) and three operating modes (Kerosene, Diesel and Naphtha runs) are combined to represent current and future oil resources and reserves, oil derivatives production and refining capacity [1]. To harmonize this with petrochemical co-production, we assume that regional historical rates of propylene and BTX recovery per total atmospheric distillation (ADU) capacity remains constant over time (FCC-o and RC-o, respectively). New refinery capacity with propylene and BTX yields 15% higher than historical were also made available in the model as a simplified and conservative approach to represent the potential increase in refinery-petrochemical integration over time, which could be achieved with e.g. high-severity FCC units and assuming that BTX availability for petrochemical use increases as its demand as a high-octane blend component in the gasoline pool decreases over time [7,10].

Several Crude-Oil-to-Chemicals (COTC) projects that primarily convert light crude into chemicals are already in stage of development or trial operation – mostly in China (Hengli Petrochemical, Zhejiang Petrochemical, Hengyi Shenghong) and Saudi Arabia (Aramco/SABIC) – with an estimated chemical conversion rate of more than 40% [11]. While an important trend in the petrochemical sector, representing COTC technologies was not possible given the lack of reliable and publicly available data. For now, we are aware that our results on future oil use for non-energy purposes may be underestimated (especially in less carbon constrained scenarios) and that refinery-petrochemical integration is a critical topic in the future role of oil and gas that should be addressed in future studies.

- Multiproduct technologies

Steam cracking (SC-Naphtha and SC-Ethane)

Steam crackers are at the heart of the petrochemical industry. They break down saturated hydrocarbon streams (ethane, propane, butane, naphtha, and gasoil) into unsaturated compounds such as ethylene, propylene, butadiene, and BTX.

The steam cracking process is a non-catalytic pyrolysis of hydrocarbons, performed under temperatures that go up to 900°C, low pressure (around 1.5 bar) and short residence time (<1s). Under those conditions, carbon-carbon and carbon-hydrogen bonds are broken to make olefins and aromatics. Steam cracking of lighter streams (i.e., ethane) result in higher relative yields for ethylene whereas heavier streams (i.e., gasoil) yield a more balanced basket of products and fuel-grade by-products (e.g., hydrogen, methane, and butanes) (Supplementary Figure 2).

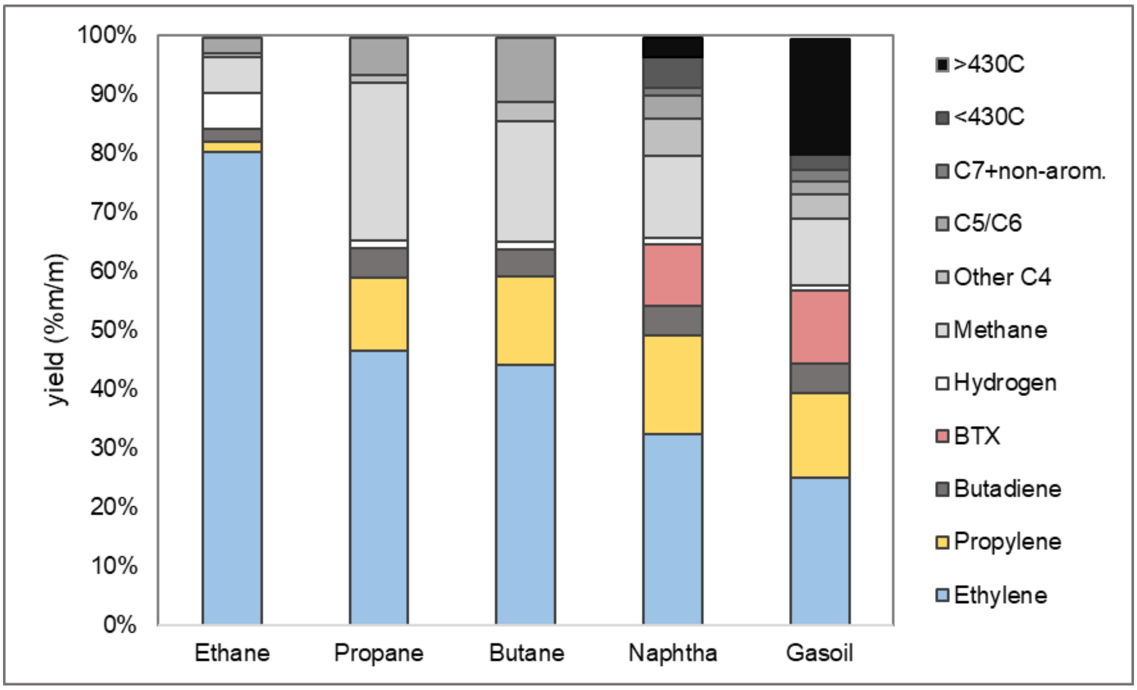

Supplementary Figure 2. Steam cracking yields in mass terms (%m/m) according to feedstock. Source: own elaboration based on [12,13]. BTX: Benzene, Toluene, Xylene (aromatic hydrocarbons); >430C: Products boiling at temperatures above 430 degrees Celsius; <430C: Products boiling at temperatures below 430 degrees Celsius; C7+ non-arom.: Non-aromatic hydrocarbons with seven or more carbon atoms; C5/C6: Hydrocarbons with five or six carbon atoms; Other C4: Hydrocarbons containing four carbon atoms other than Butadiene.

Naphtha steam cracking has traditionally been pivotal in the production of HVCs. Compared to other feedstocks, naphtha use has been more advantageous given its higher flexibility to HVCs demand and pricing fluctuations. However, over the past two decades, there has been a

transition in feedstock use towards ethane, especially in gas-rich regions. The Ethylene/Propylene ratio (E/P) resulting from ethane steam cracking is much higher than those of other feeds (Supplementary Figure 2). This has resulted in a significant deficit in propylene supply worldwide, notably in Asia. To bridge this propylene gap, several on-purpose technologies were deployed worldwide (e.g, Methanol-to-Olefins in China, and Propane Dehydrogenation in the Middle East and in the US).

Historical capacity data was sourced from the International Survey Of Ethylene From Steam Crackers [14]. Our model only included ethane and naphtha steam crackers in the model; for simplicity, we treated the capacity of propane, butane, and gasoil steam crackers as though it were from naphtha steam crackers.

Furthermore, we deem bionaphtha (e.g., co-product of HVO or SAF production via Fischer–Tropsch synthesis or oligomerization) to be chemically indistinguishable from fossil naphtha, and therefore, it can also be used in SC-Naphtha units. Also, we considered that the part of the process energy demand is met by fuel-grade by-products such as hydrogen and methane [15,16].

Naphtha catalytic cracking (NCC)

Naphtha Catalytic Cracking (NCC) is an alternative steam cracking solution to make up for propylene gap while still using naphtha as feed. While P/E ratio in traditional naphtha steam cracking is around 0.5, NCC can deliver P/E ratios up to 1 with an increase in yield for aromatics as well [17–19]. Employing zeolite catalysts enhances the overall process, boosting both the selectivity for olefins and aromatics and the efficiency of the operation. To date, a singular commercial plant utilizing the New Catalytic Cracking (NCC) process has been established in Korea. This plant mirrors a standard Fluid Catalytic Cracking (FCC) unit in design, but is specifically configured for petrochemical production [18].

- On-purpose technologies

Propane dehydrogenation (PDH)

Propane dehydrogenation (PDH) is one of the main technologies used so far to bridge the propylene supply gap, mainly in the United States, Middle East, and China. PDH has been available for decades as an alternative to produce polymer-grade propylene with a high selectivity from propane-rich streams and is usually carried out in the presence of a Platinum or Chromium catalyst under low pressure and high temperatures [20]. Bio-propane from bio-LPG

streams was also considered as a feedstock. PDH regional historical capacity data was collected from [21].

Bioethanol dehydration (BDH)

Bioethanol, the most produced biofuel globally, primarily originates from maize in the United States, sugarcane in Brazil, and sugar beet in Europe. Unlike biodiesel, bioethanol is chemically identical to its fossil fuel-based counterpart and is utilized in internal combustion engine. As the long-term demand for gasoline and ethanol for passenger transportation declines, the dehydration of bioethanol (BDH) to ethylene emerges as a promising technology to decarbonize ethylene production. In 2010, Braskem deployed a bioethanol dehydration plant with annual ethylene capacity of 200kt [22]. Furthermore, ethylene can undergo oligomerization to form bio-jet fuel, green diesel, and bio-naphtha, aligning with decarbonization strategies in traditionally challenging sectors such as aviation and shipping.

Methanol-to-olefins (MTO)

Methanol-to-olefins (MTO) is an autocatalytic reaction that converts methanol from any feedstock to ethylene and propylene, often over a zeolite catalyst. It assumed significant strategic importance in China as a viable alternative source of olefins, particularly during periods of elevated oil prices [23].

In our modelling, methanol is identified as a critical pathway through which every HVC can be sourced via Carbon Capture and Utilization (CCU) methods. Initially, CO_2 is hydrogenated to methanol (see Carbon Dioxide Hydrogenation below), which can subsequently be converted to ethylene and propylene via MTO. The resulting ethylene can undergo a sequence of dimerization and Catadiene processes to form butadiene. Finally, aromatics can be generated through the Methanol-to-Aromatics (MTA) pathway, further detailed below.

Regional historical capacity data was collected from numerous references, with a particular relevance for China. This focus is due to the significant implementation of this technology in the country, where methanol production, primarily sourced from coal, is prevalent [24–27].

Dimerization (DIM)

The dimerization (DIM) of ethylene is a chemical process that combines two ethylene molecules to produce butenes, commonly referred to as a C4 stream, which includes a mixture of both 1-butene and 2-butene isomers. In our modelling approach, we accommodate the use of alternative bio-/CCU-based feedstock, while also allowing the use of ethylene into olefin

conversion technologies. Dimerization thus enables the conversion of ethylene to propylene via dimerization followed by metathesis [28].

Metathesis (MTT)

Metathesis (MTT) is a chemical process that transforms ethylene and either 2-butene or 1-butene (referred to as a C4 stream) into propylene. In the COFFEE model, this C4 stream is primarily generated from the dimerization of ethylene, but also as a co-product of the PDH process. Metathesis reactions entails the formation and cleavage of carbon-carbon double bonds, facilitated by catalysts that typically employ ruthenium, molybdenum, or tungsten. 1-butene molecules undergo isomerization to become 2-butene, which subsequently react with ethylene in the presence of a tungsten oxide catalyst [28]. On a stoichiometric basis, this reaction yields two moles of propylene for every mole of ethylene.

Ethanol-to-Butadiene (ETB)

Ethanol-to-Butadiene (ETB) is being considered as a bio-based alternative to butadiene production based on the renewable ethanol platform. Although the reaction mechanism is still under debate, the process generally consists of ethanol dehydrogenation to acetaldehyde, which then reacts with ethanol to form butadiene [29,30].

Methanol-to-aromatics (MTA)

The methanol-to-aromatics (MTA) process is a catalytic conversion process that transforms methanol into BTX. Much similar to the MTO technology, the process involves several steps, including dehydration, dehydrogenation, and aromatization, but requires higher temperatures (500-700°C) and lower pressures (0.01-0.1 MPa). Furthermore, specialized zeolite catalysts are used to increase the selectivity to aromatics [19,25].

Catadiene© (CAT)

Catadiene© (CAT) is based on the Houdry process – a catalytic cracking process developed in the early 1940s to convert heavy oil fractions to gasoline – and consists of a catalytic dehydrogenation of n-butanes and n-butenes (C4s stream) into butadiene [6,31–33].

Catalytic Reforming unit (CR-n)

Conventional catalytic reforming is a process that converts low octane straight run naphtha into high-octane gasoline. This is performed by converting paraffinic and naphthenic compounds into aromatics, which increases the octane value of the gasoline.

An alternative option was also created to produce aromatics in the model, but outside the oil refining module. However, for this study, aromatics in this unit are produced exclusively for

petrochemical production, being fed either by fossil fuel-based or bio-based naphtha. Cost assumptions were based on Guedes (2019). These costs include not only the expenses for the process unit (inside battery limits or ISBL) but also the direct and indirect costs associated with supporting infrastructure. This includes utilities, piping, storage, and electrical installations, among others (outside battery limits or OSBL).

Methanol and ammonia

Processes for ammonia, methanol and hydrogen production are quite similar in that they all have syngas (a mixture of carbon monoxide and hydrogen) as intermediates. In the COFFEE model, syngas can be produced via steam methane reforming (SMR), coal gasification (CGS), biomass gasification (BGS), partial oil oxidation (POX). Water electrolysis and carbon dioxide hydrogenation (CDH) are alternative routes to hydrogen (as input to ammonia production) and to methanol production, respectively.

Ammonia (NH_3) is the primary chemical of all nitrogen fertilizer and one of the top 3 chemicals transported globally [35,36]. Agriculture uses cover around 70% of its global demand, and other applications span across several industrial sectors, including the production of nitric acid, an essential precursor for explosives, plastics, and polyurethanes [37]. Its conventional production method is the Haber-Bosch process, in which N_2 and H_2 react in a 1:3 ratio under high pressure and temperature. N_2 is separated from air mixture in a conventional air separation unit (cryogenic distillation/ pressure swing adsorption) and H_2 is usually sourced by steam methane reforming (SMR) followed by a water-gas shift reaction [38]. Ammonia supplied from H_2 produced by coal gasification (CGS) is particularly relevant in China and South Africa, whereas partial oxidation (POX) of heavy oil is still produced in Europe and India. Global production reached around 150 million tonnes in 2015 [39].

In ammonia production, carbon monoxide in syngas is converted to CO_2 and then either emitted or captured to be used in urea production. Therefore, given that CO_2 capture and utilisation is a common practice in ammonia-urea integrated facilities, ammonia's process emissions have been adjusted according to regional urea production available in FAOSTAT [40,41]. We assumed that the stoichiometric CO_2 demand for urea production is fulfilled by ammonia captured emissions, assuming that regional Urea/Ammonia production ratio remained constant over the century. The residual CO_2 (i.e., that which is not utilized by urea production facilities) is assumed to be released into the atmosphere as process emissions. Alternatively, if CO_2 from ammonia production is not available, urea can be synthesized using CO_2 sourced from other methods (e.g., carbon capture).

Methanol (CH_3OH), a simple alcohol with the formula CH_3OH, is a key chemical with a wide array of applications – mostly as feedstock, solvent, or fuel. It serves as a crucial feedstock for

the production of formaldehyde, acetic acid, and several other chemicals, as well as an alternative fuel in internal combustion engines [42].

Methanol is primarily produced through steam methane reforming as well, given that the production of methanol often takes place in a fertilizer plant, where the same synthesis gas is utilized to manufacture ammonia and urea. Coal gasification processes are also relevant, particularly in China and South Africa. While ammonia synthesis requires a highly pure hydrogen stream, methanol production utilizes syngas in a 2:1 H_2/CO ratio, which leads to considerably lower process emissions in the latter.

Global methanol production increased from 50 million tonnes in 2010 to 100 million tonnes in 2019, approximately [43]. The primary drivers of demand growth have been the expansion of Methanol-to-Olefins (MTO) facilities and the introduction of standards for blending methanol into gasoline (ranging from M5 to M100), predominantly occurring in China. Methanol is also quite relevantly sourced from coke oven gas facilities integrated to steel production in China [44]. This information was incorporated into the steel module in COFFEE. We added a technology that simultaneously generates methanol and steel, taking into account technoeconomic data cited in references [45–47].

Data regarding historical regional production capacity and the technology split for methanol and ammonia were gathered from a variety of sources, including company reports [48,49], scientific articles [35,44] and associations and government reports [37,40,43,50–53].

Below, we describe the technologies incorporated into the COFFEE model to improve the representation of methanol and ammonia production. We present steam methane reforming, coal gasification, partial oil oxidation, and biomass gasification as syngas-based technologies, as well as carbon dioxide hydrogenation (for methanol) and electrolysis (for ammonia) as hydrogen/electricity-based technologies. Lastly, the respective methanol and ammonia synthesis are described.

- Synthesis gas production routes

Steam methane reforming (SMR) is well-established process that converts methane into syngas through a catalytic reaction with steam under temperature and pressure at approximately 700-1000°C and 25 bar, respectively. The heat required for this endothermic reaction is provided by an external source [42]. As opposed to using light sulphur-free hydrocarbons such as methane, the conversion of heavier hydrocarbons such as coal into

syngas requires higher temperatures (i.e., up to 1800°C) that make the catalytic pathway unfeasible. As a result, **coal gasification (CGS)** has been prioritized in countries with vast coal reserves and where the transportation of natural gas is not economical, such as China and South Africa. It is a process in which pulverized coal is mixed with a gasifying agent such as steam, air, or oxygen to produce syngas. The high temperature syngas follows steps of cooling, which sometimes happens with heat recovery to achieve better energy efficiency, and clean up to remove sulphur, ammonia, mercury and other contaminants as well as particulate matter. Furthermore, gasification is usually the process to handle solid hydrocarbons, which makes **Biomass gasification (BGS)** a similar but renewable alternative to produce syngas. Lastly, **partial oxidation (POX)** aims to react heavy liquid hydrocarbon feedstocks – such as fuel oil and petroleum residues – with sub-stoichiometric oxygen. Its H_2 to CO ratio is lower than SMR (which is around 3-4) but higher than coal/biomass gasification (around 0.3-0.7).

Given the similar initial steps in producing syngas, methanol and ammonia production share are often integrated to benefit from synergies like shared infrastructure and energy efficiency, for example.

- Carbon Capture and Storage (CCS) technologies

We use CGS, SMR and BGS technologies integrated with carbon capture and storage (CCS) based on the amine-based chemical absorption. This is a well-established method currently used to capture CO_2 to produce urea in integrated facilities.

- Electrolysis/Carbon dioxide Hydrogenation

Electrolysis and Carbon Dioxide Hydrogenation (CDH) are alternatives to syngas-based technologies to produce hydrogen for ammonia production and methanol, respectively.

Electrolysis refers to the splitting of the water molecule into H_2 and O_2 with electricity. In COFFEE, electricity is supplied to this technology directly from the electricity generation module of each region, thus it can include both renewable and non-renewable electricity. Furthermore, we assume that O_2 is vented for simplification purposes, thus incomes from potential revenues or use in oxy-fuel or oxidation processes are not accounted for.

Current electrolysis technologies considered for large-scale deployment are: i) Alkaline electrolysis, which is well-established and cost-effective but requires high-quality water and is less efficient (50-78%); ii) anion-exchange membrane (AEM), which can utilize various water

types but is still under development and is more expensive; iii) proton-exchange membrane (PEM) electrolysis, an alternative highly efficient and adaptable to changing power inputs, but it requires pure water and is costly; and iv) solid oxide electrolysis, which present high efficiency (>85%) and has high operating temperatures but also presents low TRL [54,55]. In COFFEE, we use technoeconomic data available for the PEM electrolysis to represent this technology.

Carbon dioxide hydrogenation converts CO_2 into methanol via hydrogenation followed by reversed WGS reactions. In the COFFEE model, CDH is the key CCU pathway to produce carbon-bearing primary chemicals. In COFFEE, hydrogen inputs to CDH can be sourced by any of the technologies above (SMR, CGS, POX, their CCS counterparts or Electrolysis) and CO_2 is likewise supplied by any carbon capture technology across the model.

- Haber-Bosch synthesis and methanol synthesis

The syngas produced via SMR, CGS, POX, or BGS is followed by the water gas shift (WGS) reaction, during which CO and H_2O are converted to CO_2 and H_2.

For the methanol synthesis, this step has the role of adjusting the syngas composition to the optimal CO/H_2 ratio for methanol synthesis through catalytic hydrogenation using a copper-based catalyst under approximately 300°C and 2000-3000MPa.

In contrast, for ammonia production, WGS is designed to maximize the output of hydrogen. The excesses of CO_2 and CO are removed via chemical absorption and methanation, respectively, preventing the poisoning of the Haber-Bosch synthesis catalyst. After purification, H_2 is mixed with nitrogen in a high pressure synthesis over an iron catalyst to produce ammonia (Haber-Bosch synthesis). This step is the same followed by hydrogen production via water electrolysis.

Other chemicals

To achieve a comprehensive representation of the chemical sector (encompassing energy use and CO_2 emissions from both organic and inorganic chemicals) in the COFFEE model, we used data from the International Energy Agency's World Energy Balances[56]. By doing this, we accounted for the regional energy use gap between the production of primary chemicals and the remainder of the chemicals sector. We integrated heat and machine drive production technologies into the model to bridge this gap, thereby facilitating fuel switching, electrification, and efficiency improvements over time.

Supplementary Method 3. Technoeconomic parametrization of chemical processes

Supplementary Table 1, Supplementary Table *2*, and Supplementary Table 3 detail the technoeconomic parameters for the production of HVCs, methanol, and ammonia, respectively. Primary references were used for refinery[34,57] and chemical processing[19,47,58] units data, which were validated and complemented drawing on multiple sources.

To ensure consistency and comparability across processes, we standardized all values to the same units and currency. This process included the conversion of economic and physical units. For economic conversions, the Chemical Engineering Plant Cost Index (CEPCI)[59] was used to adjust the investment costs of chemical plants to 2010 US dollars. This accounts for inflation and variations in the costs of equipment, materials, and labour over time. Moreover, due to the diversity in original data – often presented in various currencies and/or from different years – we performed extensive conversions and validations.

For physical conversions, we used densities, heat of combustion, and basic unit conversion for handling material and energy efficiencies. When using heat of combustion, we applied the Higher Heating Value (HHV) when steam was explicitly included in the exhaust gases, capturing the total energy released, including the latent heat of vaporization of water. Conversely, we used the Lower Heating Value (LHV) when steam was not explicitly represented, which omits the energy associated with water vapor condensation. This differentiation ensures accuracy in energy conversion calculations depending on the presence of steam in the combustion exhaust.

In our analysis, we treated units that process bio-based, CCU-based, and fossil-based feedstocks as equivalent. For example, we assumed that catalytic reforming units convert both naphtha and bio-naphtha with identical efficiency. Similarly, whether derived from fossil, CCU or bio-based processes, methanol is chemically identical. Therefore, we maintained consistent costs and yields across all these units regardless of the feedstock origin.

Supplementary Table 1. Techno-economic parameters of HVC production technologies.

Technology	Feedstock	Output yields						SEC[a]	Plant capacity	Investment cost	O&M cost		Ref.
		Ethylene	Propylene	Butadiene	BTX	C4 stream	H_2				FOM	VOM	
		t/t_F						GJ/GJ_F	kt_{MO}/yr	USD2010/ $t_{MO}py$[b]	USD2010/t_{MO}		
SC-Naphtha	Naphtha	**0.324**	0.168	0.050	0.104			10	500	2718	54	54	16,19,60–62
SC-Naphtha-b	Bionaphtha	***0.324***	*0.168*	*0.050*	*0.104*			10	500				
SC-NG	Ethane	**0.803**	0.016	0.023	0			15	500	1680	34	34	
NCC	Naphtha	**0.324**	0.324	0.050	0.130			9.3	400	3963	41	41	19,60,63,18
NCC-b	Bionaphtha	***0.324***	*0.324*	*0.050*	*0.130*			9.3	400				
BDH	Ethanol	***0.575***						2.8	200	1190	84	84	
MTO	Methanol[c]	**0.190**	0.180					4.3	500	1340	34	34	19,60,63,18,24,64,26,27,65
MTO-b	Biomethanol	***0.190***	*0.180*					4.3	500				
PDH	Propane		**0.750**			0.19		9.1	500	855	23	23	19,60,20,32
PDH-b	Biopropane		***0.750***			*0.19*		9.1	500				
MTT	Ethylene (+ C4s)[d]		**3.125**					3.6	300	750	44	44	28,66
MTT-b	Bioethylene (+ bC4s)[d]		***3.125***					3.6	300				
ETB	Ethanol			***0.280***				21.5	50	800	67	23	29
MTA	Methanol[c]				**0.230**			3.8	500	1380	17	17	19,60,64,27
MTA-b	Biomethanol				***0.230***			3.8	500				
CAT	C4 stream			**0.600**				11.4	500	855	23	23	19,60,20,32
CAT-b	Bio C4 stream			***0.600***				11.4	500				
DIM	Ethylene					**0.800**		0.4	300	150	4	4	28
DIM-b	Bioethylene					***0.800***		0.4	300				
CR	Naphtha				**0.880**		3.6[e]	0.8	800	262	3	3	34,57
CR	Bionaphtha				***0.880***		3.6[e]	0.8	800	262	3	3	34,57

F: Feedstock (underlined); **MO: Main output (values in bold).** *Bioproducts in italic.* [a] Includes heat, steam, and machine drive requirements (thus excluding energy use as feedstock). [b] Tonnes per year. [c] Fossil-. or CCU-based. [d] Yields refer to 1t of ethylene and 3.03t of C4s. [e] Value in GJ/t_{MI}. SC: Steam cracking; NCC: Naphtha catalytic cracking; BDH: Bioethanol dehydration; MTO: Methanol-to-Olefins; PDH: Propane dehydrogenation; MTT: Metathesis; ETB: Ethanol-to-Butadiene; MTA: Methanol-to-Aromatics; CAT: Catadiene®; DIM: Dimerization; CR: Catalytic reforming; Suffix *-b*: bio-based feedstock and products.

Supplementary Table 2. Techno-economic parameters for methanol production technologies.

Technology	Feedstock	Yields		Machine drive	Process Emissions	Investment cost	O&M cost		Ref.
		MO: Methanol	CO_2 *capture*/**use**				FOM	VOM	
		GJ_F [a] /t_{MO}	tCO_2/t_{MO}	GJ/t_{MO}	tCO_2 /t_{MO}	USD2010/t_{MO}py [b]	USD2010/t_{MO}		
SMR	Methane	33.9	-	0.3	0.8	340	4.5	4.5	[19,47,58]
SMR+CCS	Methane	33.9	*0.76*	0.3	0.04	540	6.7	6.7	[19,47,58]
CGS	Coal	46.3	-	3.7	3.3	820	10.3	10.3	[19,47,58]
CGS+CCS	Coal	55.3	*3.14*	3.9	0.17	1020	12.8	12.8	[19,47,58]
BGS	Solid biomass	47.9	-	5.0	0	5655	70.7	70.7	[19,47,58,67]
BGS+CCS	Solid biomass	47.9	*3.25*	5.0	0	5855	73.3	73.3	[19,47,58,67]
CDH	H_2 + CO_2	22.6	**1.38**	1.5	0	44	0.5	0.5	[19,43,47,58]

F: Feedstock (underlined); MO: Main Output; FOM: Fixed operation and maintenance cost; VOM: Variable operation and maintenance cost. [a] Includes heat and feedstock requirements. [b] Tonnes of main output per year.

Supplementary Table 3. Techno-economic assumptions for hydrogen and ammonia production technologies. SMR, SMR+CCS, CGS, CGS+CCS, POX, BGS, BGS+CCS, and Electrolysis are hydrogen production technologies, which can be used for energy and non-energy purposes (i.e., in ammonia production via Haber-Bosch reaction).

Technology	Feedstock Inputs	Yields		Machine drive	Process Emissions	Investment cost	O&M cost		Ref.
		MO: H_2 or **NH_3**	CO_2 capture				FOM	VOM	
		GJ_{MO} [a] /GJ_{MI} or **t_{MO}/GJ_{MI}**	tCO_2/t_{MO}	GJ/t_{MO}	tCO_2 /t_{MO}	USD2010/kWyr$_{MO}$py [b]	USD2010/kWyr$_{MO}$		
SMR	Methane	0.74	-	-	8.90	545	12	12	[19,47,58,68]
SMR+CCS	Methane	0.74	8.01	-	0.89	622	23	23	[19,47,58,68,69]
CGS	Coal	0.59	-	-	20.2	2086	20	20	[19,47,58,68]
CGS+CCS	Coal	0.59	18.18	-	2.02	2255	22	22	[19,47,58,68,69]
POX	Fuel oil	0.70	-	-	13.6	800	10	10	[19,47,58,68]
BGS	Solid biomass	0.67	-	-	-	2285	83	83	[19,47,58,67,68]
BGS+CCS	Solid biomass	0.67	16.4	-	-	2465	83	83	[19,47,58,67,68]
Electrolysis	Electricity + H_2O	0.64 (2010)<br>0.74 (2060)	**-**	-	-	896 (2010)<br>240 (2050)	12 (2010)<br>3 (2050)	12 (2010)<br>3 (2050) [c]	[19,43,47,55,58,68]
Haber Bosch	H_2 + N_2	**0.04**	**-**	3.9	-	95	2.5	2.5	[19]

MI: Main Input (underlined); MO: Main Output; FOM: Fixed operation and maintenance cost; VOM: Variable operation and maintenance cost. [a] Includes heat and feedstock requirements. [b] tonnes per year. [c] Electrolysis VOM does not include potential revenues from oxygen sales.

Supplementary Method 4. Regional demand scenarios

This section delves into the methodology used to model demand scenarios across regions, taking an econometric approach to provide a general understanding of scale and rate of future petrochemicals demand. We discuss these methodologies in detail in the following subsections.

<u>High-value chemicals</u>

The production of resin and fibres are key factors that drive the demand for high-value chemicals (HVC). Thus, to calculate the long term demands for each HVC, we initially relied on per capita plastics consumption data specific to 63 countries [70]. This data was used as a proxy to estimate total plastic demands from 2010 to 2100 for the 18 regions in COFFEE, described in Supplementary Table 4. The historical data used in this study is illustrated in Supplementary Figure 3 .

Subsequently, we conducted a regression analysis to project future demand. We incorporated data on GDP and population data according to the second shared socioeconomic pathway (SSP2) [71]. We use a simple modified form of exponential growth model (Supplementary Equation 1) based on macroeconomic assumptions to project long-term demands:

$$Cpc_t = Cpc_{t-1} \cdot \left(\frac{GDPpc_t}{GDPpc_{t-1}}\right)^{\frac{ln\left(\frac{Cpc_{2015}}{Cpc_{2010}}\right)}{ln\left(\frac{GDPpc_{2015}}{GDPpc_{2010}}\right)}} \quad (1)$$

where:

Cpc_t: Plastics Consumption per capita at timestep t

Cpc_{t-1}: Plastics Consumption per capita at the previous timestep $t-1$

$GDPpc_t$: Gross Domestic Product per capita at timestep t

$GDPpc_{t-1}$: Gross Domestic Product per capita at the previous timestep $t-1$.

Cpc_{2010}, Cpc_{2015}, $GDPpc_{2010}$, and $GDPpc_{2010}$: The respective Plastics Consumption per capita and Gross Domestic Product per capita in the timesteps 2010 and 2015.

The model uses the ratio of growth rates between the current year and the previous year to adjust the rate of growth changes over time. Additionally, the logarithmic term normalizes the projection based on historical trends by comparing the ratio of plastic consumption per capita in a base year (i.e., 2015) to a prior year (i.e., 2010) with the ratio of growth rates over the same period. Due to low availability data, we decided to undertake this conservative approach to

estimate future consumption per capita (i.e., it assumes that plastic consumption will continue to grow at a rate proportional to its current level, and that the rate of growth will be constant over time).

We compared the first estimates with other studies[72–74] and reached the conclusion that: 1) the primary data for plastics per capita consumption had a reduced scope compared to the assessment made by [19,73], and 2) HVCs are also used in other applications such as solvents, surfactants and detergents. Thus, to account for this "other" component of HVC demand, we normalized global demands based in the figures provided by Geyer et al. (2017). We then calculated the content of ethylene, propylene, butadiene, and BTX in 1 kg of plastic, based on figures from a material flow analysis work of 2013 [74,75]. These shares were assumed to remain constant over time.

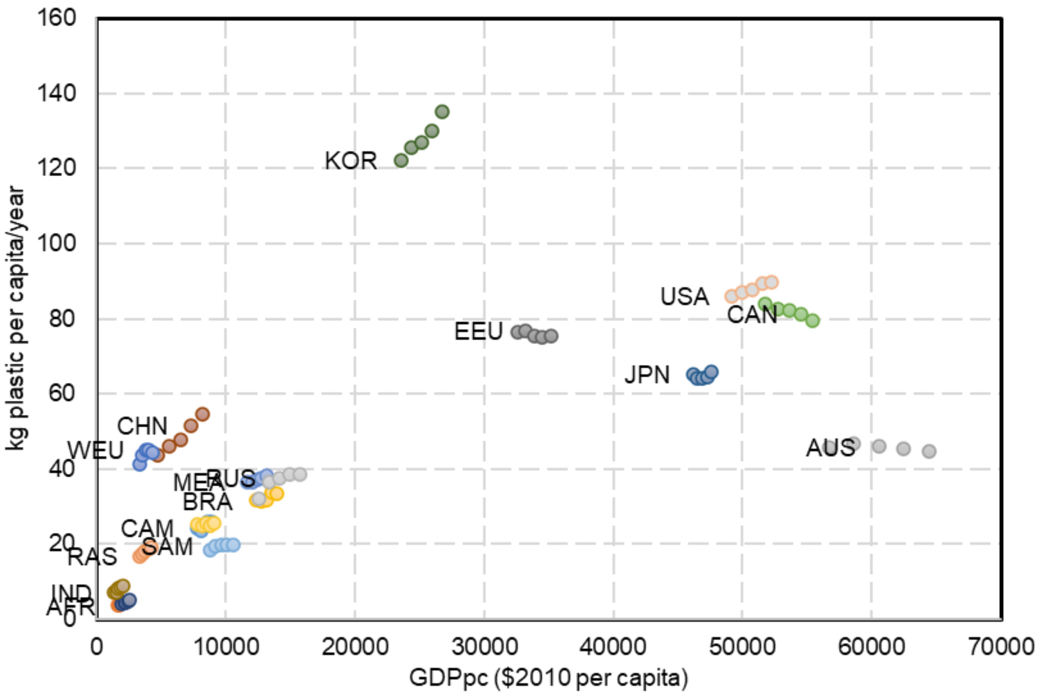

Supplementary Figure 3. Development of per capita consumption of plastics per COFFEE region based on ref.[70] . Plastics comprise thermoplastics such as PVC (Polyvinyl Chloride), PE (Polyethylene), including HD-PE (High-Density Polyethylene), LD-PE (Low-Density Polyethylene), and LLD-PE (Linear Low-Density Polyethylene), PP (Polypropylene), PS (Polystyrene) which includes GP (General Purpose Polystyrene) and HI (High Impact Polystyrene), ABS (Acrylonitrile Butadiene Styrene), SAN (Styrene Acrylonitrile), PET Resin (Polyethylene Terephthalate Resin), PA (Polyamide) which includes PA6 (Polyamide 6 or Nylon 6) and PA66 (Polyamide 66 or Nylon 66), and PC (Polycarbonate). Data shows increasing levels of plastics consumption in regions with higher GDPpc. GDPpc: Gross Domestic Product per capita. $2010: United States dollar in 2010; AFR: Africa (excl. South Africa); AUS: Australia and New Zealand; BRA: Brazil; CAM: Central America; CAN: Canada; CAS: Caspian Region; CHN: China; EEU: European Union (28); IND: India; JPN: Japan; KOR: South Korea; MEA: Middle East; RAS: Rest of Asia and Oceania; RUS: Russia; SAF: South Africa; SAM: South America (excl. Brazil); USA: United States; WEU: Other Europe.

Supplementary Table 4. Description of regions in COFFEE.

Tag	Description
**AFR**	Africa (excl. South Africa)
**AUS**	Australia and New Zealand
**BRA**	Brazil
**CAM**	Central America
**CAN**	Canada
**CAS**	Caspian Region
**CHN**	China
**EEU**	European Union (28)
**IND**	India
**JPN**	Japan
**KOR**	South Korea
**MEA**	Middle East
**RAS**	Rest of Asia and Oceania
**RUS**	Russia
**SAF**	South Africa
**SAM**	South America (excl. Brazil)
**USA**	United States
**WEU**	Other Europe

Methanol and Ammonia

A similar approach was followed to project methanol long-term regional demands. Given that methanol use in biodiesel production and gasoline blending was already endogenously represented in COFFEE, we model methanol demand for non-energy purposes separately, e.g., formaldehyde, acetic acid, and methylamine production. To that end, data on regional methanol demands were collected from EPE (2019) and Su et al. (2013) [44,52], from which it was reduced the energy-related demands based on Chatterton (2018) and OECD & FAO (2021) [76,77]. In the case of ammonia, we used long-term regional demands for food outputs the TEA (Total-Economy Assessment) model, which is a multi-regional and multi-sectorial Computable General Equilibrium (CGE) model used in soft-link with COFFEE[78,79]. Future studies aim to make ammonia demand endogenous with the land-use module in COFFEE.

Supplementary Method 5. Final disposal assumptions

Solid waste management data from World Bank's *What a Waste 2.0* database was used as a baseline to estimate regional shares for incineration, landfilling (i.e., controlled landfilling plus mismanagement) and mechanical recycling (see Supplementary Figure 4). Incineration and recycling emissions were calculated based on ref.[80]. For plastics incineration, it was assumed that the carbon content of each HVC was emitted as CO_2. Therefore, 3.14 tCO_2/t ethylene, 3.14 tCO_2/t propylene, 3.25 tCO_2/t butadiene, and 3.38 tCO_2/t aromatics (using benzene as a proxy) were assumed when accounting for those emissions. Mechanical recycling considers 10% of material loss at every cycle and emissions account for electricity use from shredding, extrusion, and agglomeration.

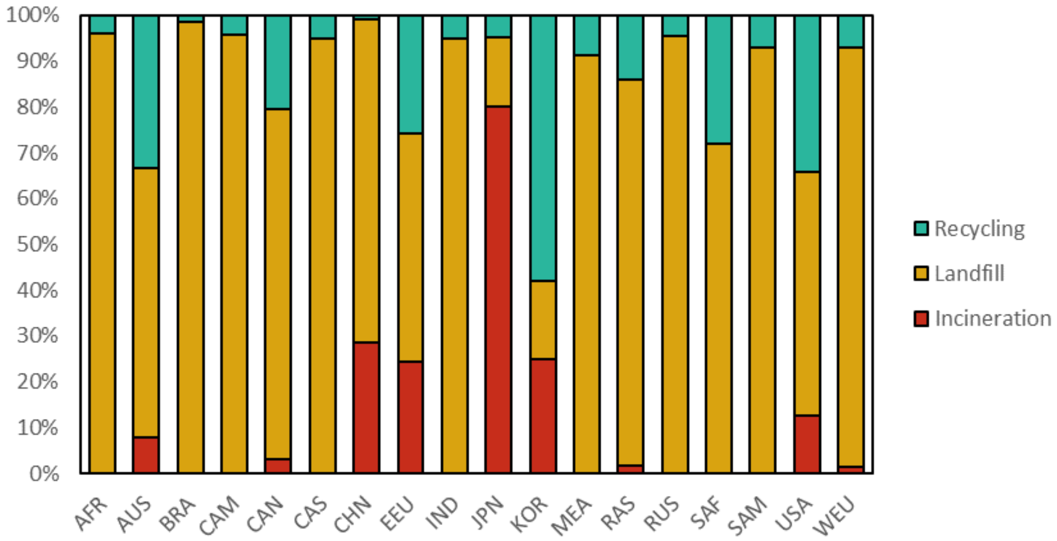

Supplementary Figure 4. Final disposal shares assumed in the base year for COFFEE regions. Source: own elaboration based on ref.[81].

Supplementary Method 6. Land-use emissions considerations

COFFEE assumes carbon neutrality for biomass based on specific conditions. Firstly, the model includes emissions from land-use changes, energy use during agricultural practices and logistics, agricultural residues, fertilizer use, and other relevant sources. It also considers carbon sequestration during biomass growth and the final storage of carbon, either geologically or in materials. Within the balance between carbon emissions and sinks, the model identifies and favours options and agriculture practices that guarantee net negative carbon emissions.

COFFEE represents Sugarcane, Corn, Wheat, Woody biomass, Grassy biomass, Beet, Bagasse, Residues. It considers the growth rates and carbon uptake capacities of different species to ensure that the biomass used has a high potential for carbon neutrality. Harvest methods are not explicitly represented, but are indirectly assumed via crop yields, energy inputs, capital investment and operating costs (incl. labour). The model does not account for soil carbon changes due to biomass harvesting. Soil carbon is affected only through land-use change.

More information on the land-use sector in COFFEE can be found in refs.[82–85].

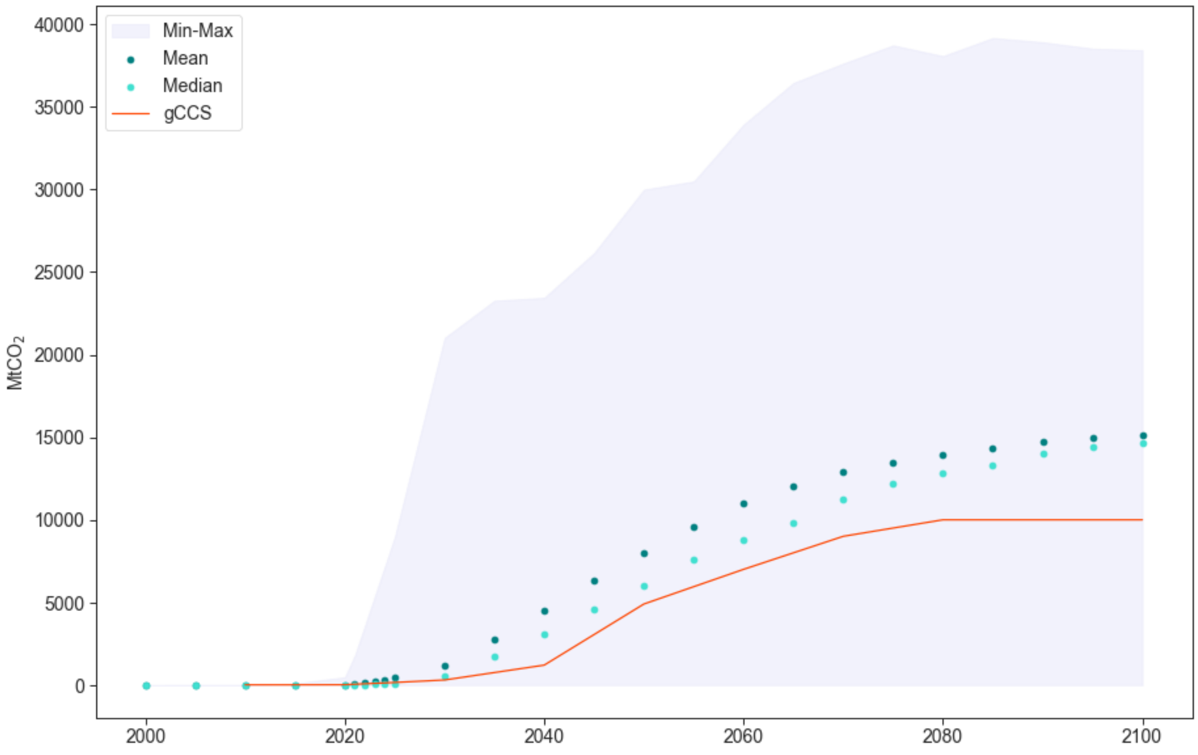

Supplementary Figure 5. Assumptions concerning the global deployment of Carbon Capture and Storage (CCS) in 1p5C_gCCS scenario compared with outcomes derived from 540 scenarios across categories C1, C2, and C3 from the IPCC AR6 scenario database. gCCS: a 1.5C scenario with restrictions on global CCS deployment.

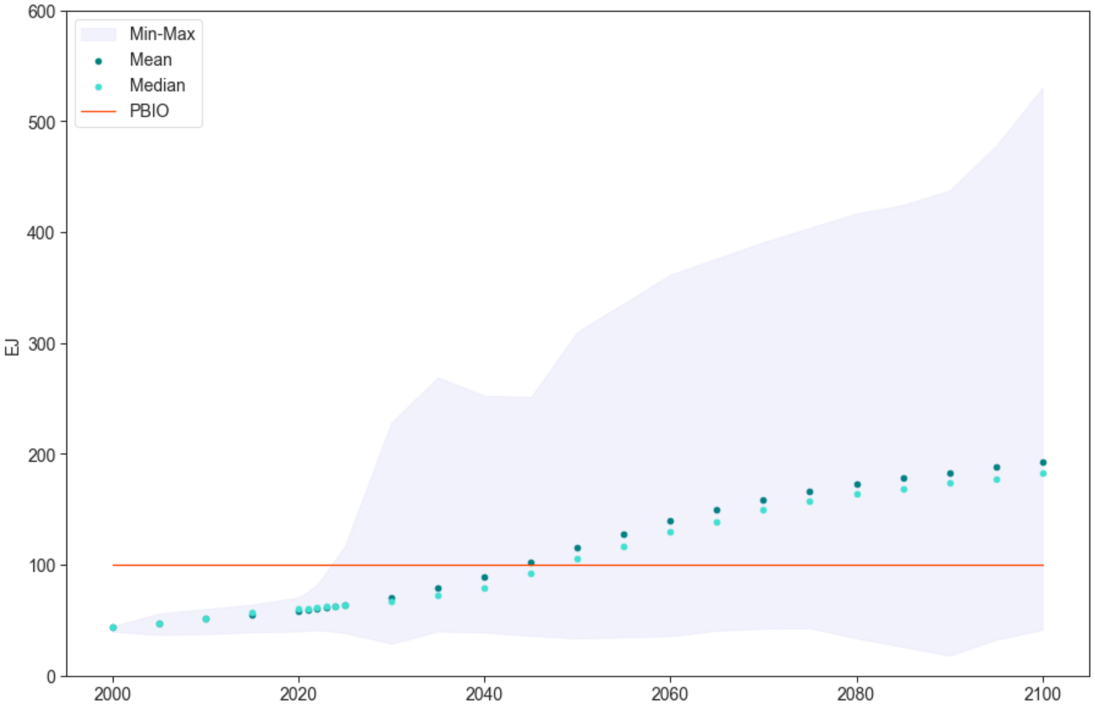

Supplementary Figure 6. Assumptions concerning the utilization of global biomass as a primary energy source in 1p5C_PBIO scenario compared with outcomes derived from 540 scenarios across categories C1, C2, and C3 from the Intergovernmental Panel on Climate Change (IPCC) Sixth Assessment Report (AR6) scenario database. PBIO: a 1.5C scenario with constraints on global primary biomass use.

Supplementary Table 5. Scenario definition and constraints assumptions.

					Global CCS restriction, reaches 10 Gt/yr in 2080	Biogenic carbon storage in biomaterials	Restricts biomass use below 100 EJ/yr globally after 2040
	$GtCO_2$		AR6 WGI		COFFEE constraints		
Scenario / Period	Budget COFFEE[a] (2018-2100)	Budget AR6 WGI[b] (2020-2100)	T_L (∘C)	Likelihood	gCCS	MNET	PBIO
NPi	-	-	-	-	off	on	off
1p5C	480	400	1.5	67%	off	on	off
sensitivity analysis							
1p5C_gCCS	480	400	1.5	67%	on	on	off
1p5C_MNEToff					off	off	off
1p5C_PBIO					off	on	on
1p5C_all					on	off	on

T_L: Temperature increase above pre-industrial levels limit. [a] The carbon budget variable in COFFEE starts in 2018. To account for the 2018-2020 gap, ~80 $GtCO_2$ was integrated into the COFFEE model's carbon budget based on ref. [86]. [b] Source: [87].

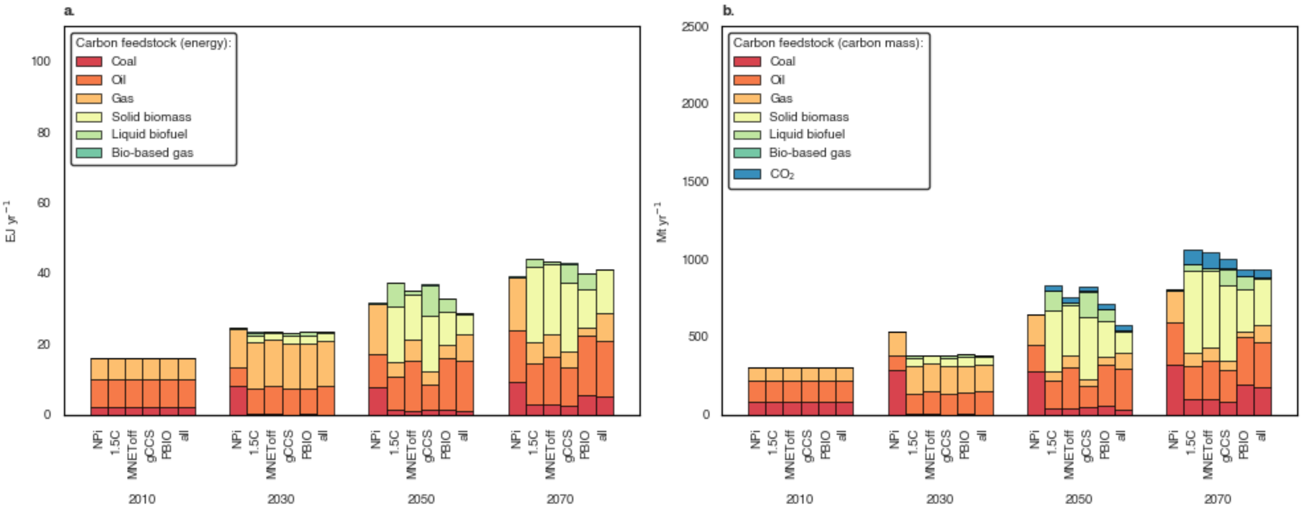

Supplementary Figure 7. Carbon feedstock in energy (a) and carbon mass (b) terms. We considered the following carbon contents of each feedtock source: Coal: 85% (on average, considering bituminous coal with high carbon content); Oil: 84.2%, based on naphtha (C_8H_{18} as a proxy); Gas: 75.0%, based on methane (CH_4) Solid Biomass: 44.7%, based on eucalyptus (see ref.[88]) Liquid Biomass: 52.2% based on bio-ethanol (C_2H_5OH); Bio-based gas: 75%, based on biomethane (CH_4); and CO_2: 27.3%. NPi: Implemented National Policies; 1.5C: carbon budget consistent with limiting global warming to 1.5°C; gCCS: a 1.5C scenario with restrictions on global CCS deployment; PBIO: a 1.5C scenario with constraints on global primary biomass use; MNEToff: a 1.5C scenario that turns off the assumption of biogenic carbon storage in materials; and all: a comprehensive 1.5C sensitivity scenario incorporating all the abovementioned restrictions.

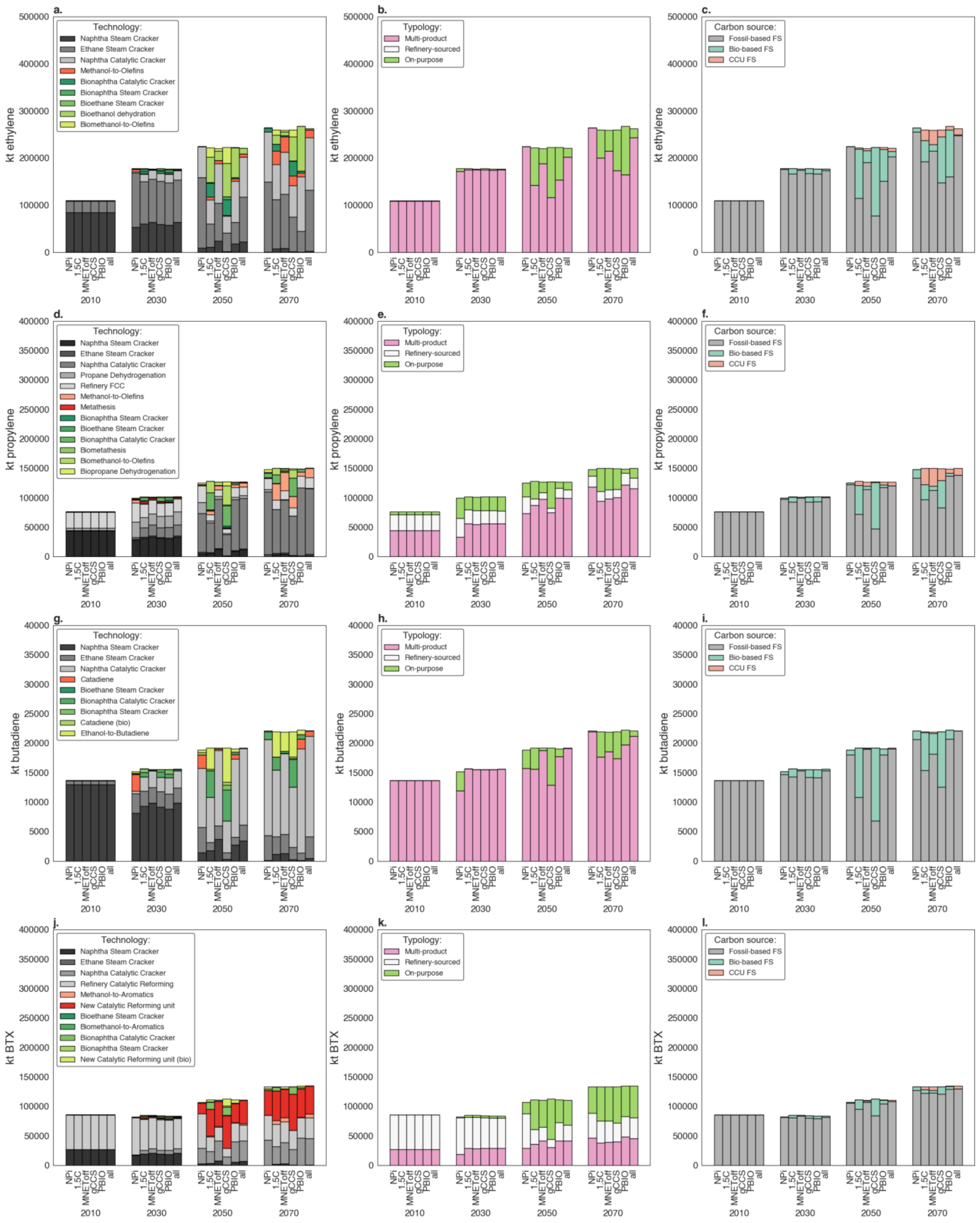

Supplementary Figure 8. Technology split, typology and carbon source for ethylene (a-c), propylene (d-f), butadiene (g-i), and Benzene, Toluene and Xylenes (BTX) (j-l) production. NPi: Implemented National Policies; 1.5C: carbon budget consistent with limiting global warming to 1.5°C; gCCS: a 1.5C scenario with restrictions on global CCS deployment; PBIO: a 1.5C scenario with constraints on global primary biomass use; MNEToff: a 1.5C scenario that turns off the assumption of biogenic carbon storage in

materials; and all: a comprehensive 1.5C sensitivity scenario incorporating all the abovementioned restrictions.

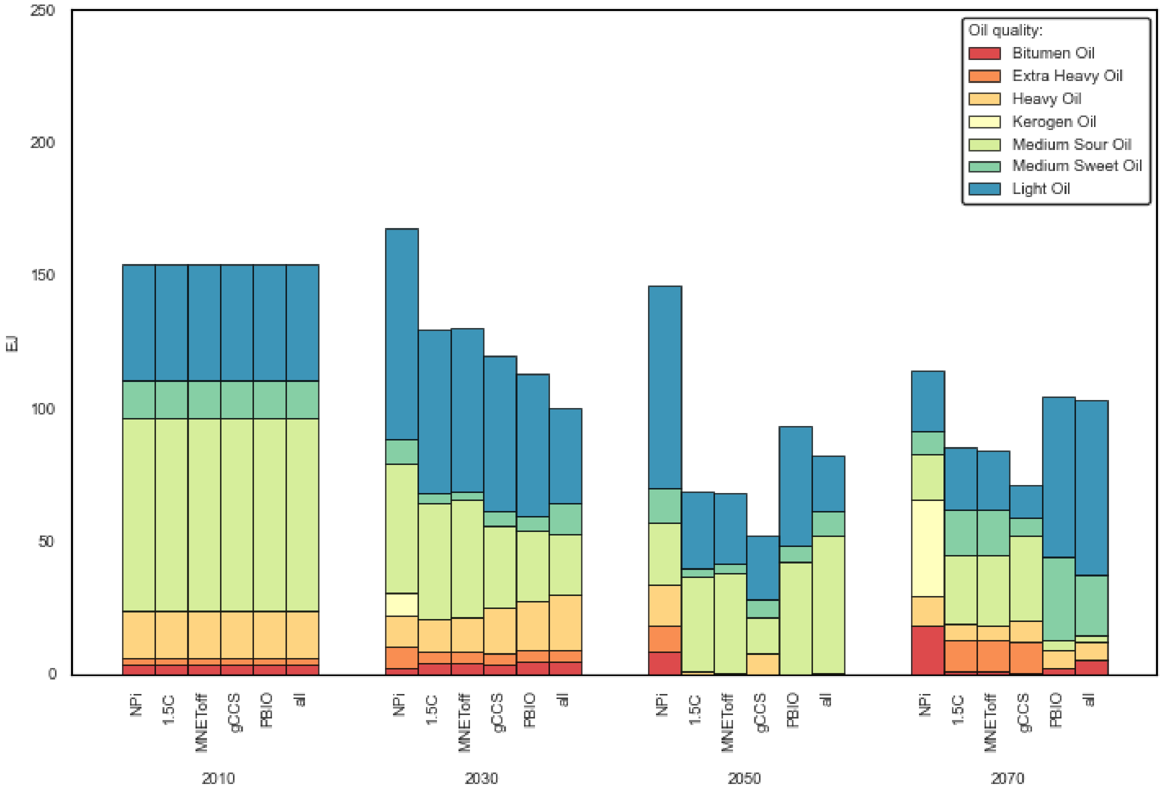

Supplementary Figure 9. Global oil production disaggregated according to oil quality. NPi: Implemented National Policies; 1.5C: carbon budget consistent with limiting global warming to 1.5°C; gCCS: a 1.5C scenario with restrictions on global CCS deployment; PBIO: a 1.5C scenario with constraints on global primary biomass use; MNEToff: a 1.5C scenario that turns off the assumption of biogenic carbon storage in materials; and all: a comprehensive 1.5C sensitivity scenario incorporating all the abovementioned restrictions.

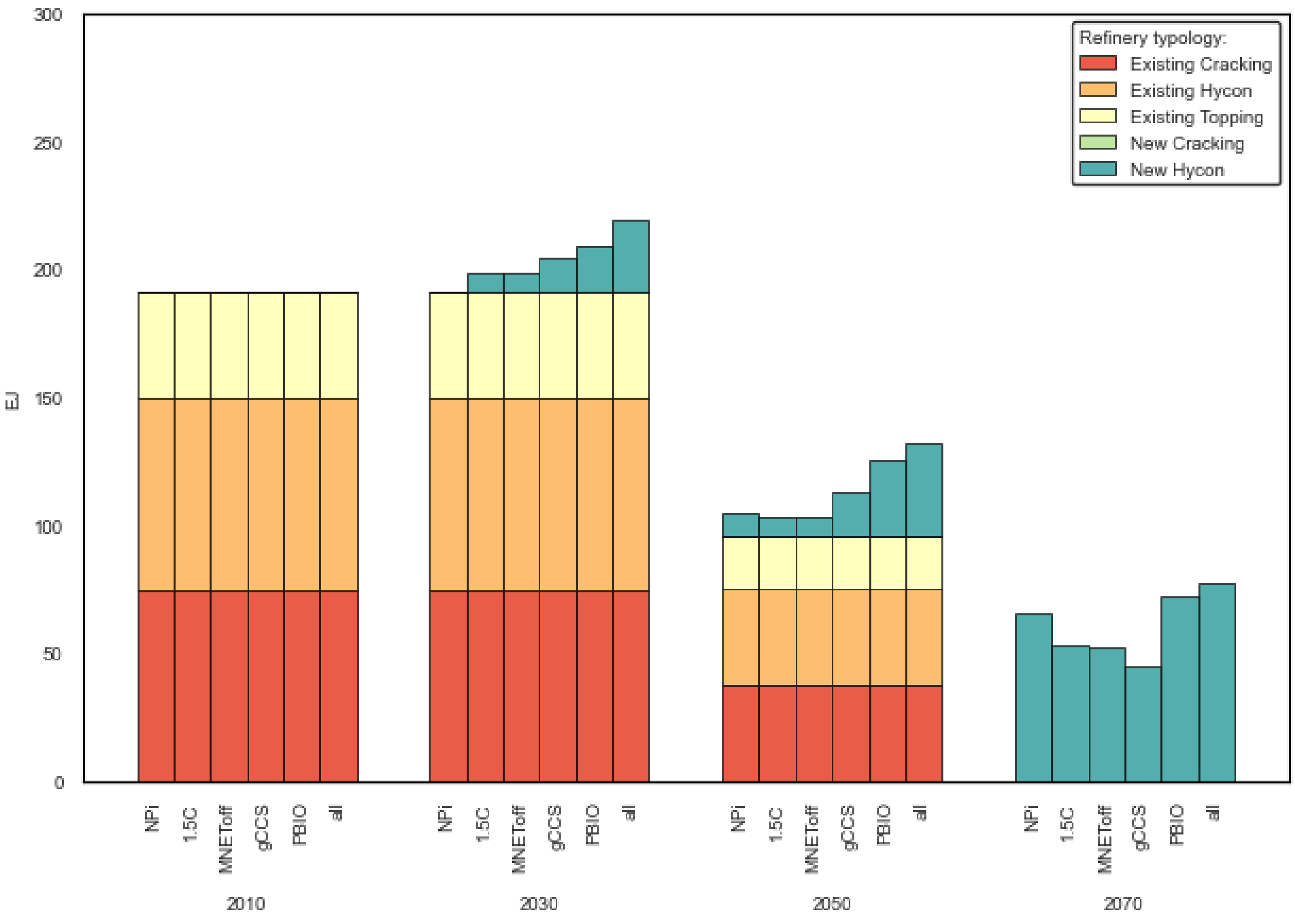

Supplementary Figure 10. Global refinery capacity per refinery typology. NPi: Implemented National Policies; 1.5C: carbon budget consistent with limiting global warming to 1.5°C; gCCS: a 1.5C scenario with restrictions on global CCS deployment; PBIO: a 1.5C scenario with constraints on global primary biomass use; MNEToff: a 1.5C scenario that turns off the assumption of biogenic carbon storage in materials; and all: a comprehensive 1.5C sensitivity scenario incorporating all the abovementioned restrictions.

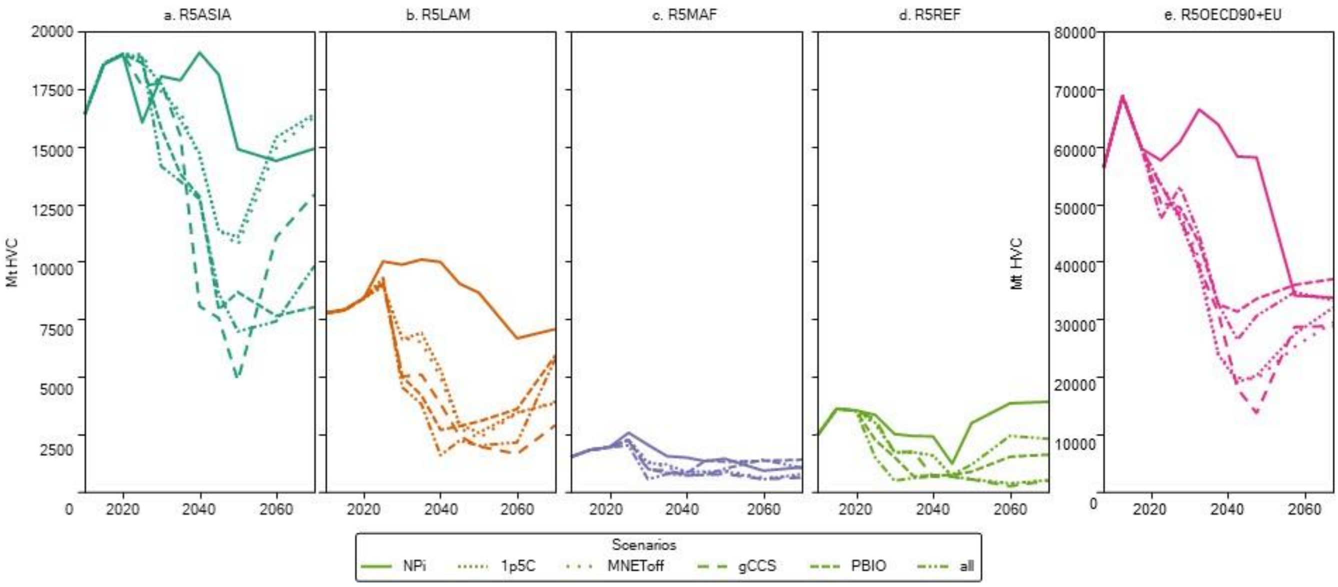

Supplementary Figure 11. Regional refinery-sourced HVCs per R5 region. a. R5ASIA: Asia (excluding Middle East); b. R5LAM: Latin America; c. R5MAF: Middle East and Africa; d. R5REF: Reforming Economies (Eastern Europe, former Soviet Union), e. R5OECD90+EU: OECD countries. NPi: Implemented National Policies; 1.5C: carbon budget consistent with limiting global warming to 1.5°C; gCCS: a 1.5C scenario with restrictions on global CCS deployment; PBIO: a 1.5C scenario with constraints on global primary biomass use; MNEToff: a 1.5C scenario that turns off the assumption of biogenic carbon storage in materials; and all: a comprehensive 1.5C sensitivity scenario incorporating all the abovementioned restrictions.

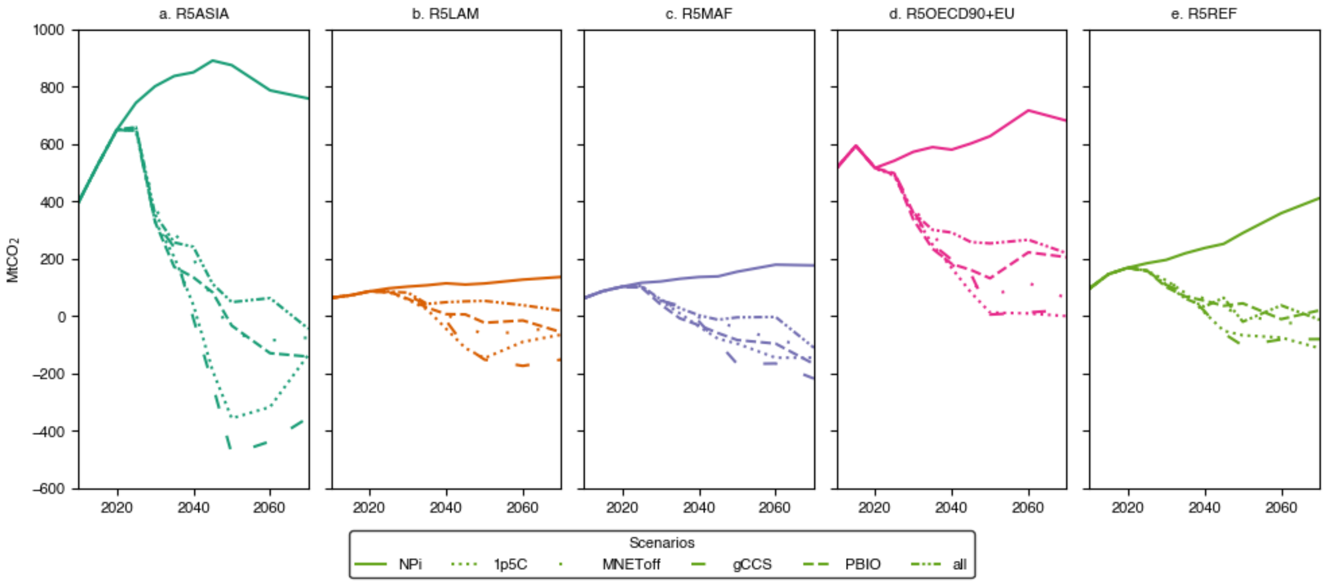

Supplementary Figure 12. Regional CO_2 emissions pathways for the chemical sector per R5 region. a. R5ASIA: Asia (excluding Middle East); b. R5LAM: Latin America; c. R5MAF: Middle East and Africa; d. R5OECD90+EU: OECD countries; e. R5REF: Reforming Economies (Eastern Europe, former Soviet Union). NPi: Implemented National Policies; 1.5C: carbon budget consistent with limiting global warming to 1.5°C; gCCS: a 1.5C scenario with restrictions on global CCS deployment; PBIO: a 1.5C scenario with constraints on global primary biomass use; MNEToff: a 1.5C scenario that turns off the assumption of biogenic carbon storage in materials; and all: a comprehensive 1.5C sensitivity scenario incorporating all the abovementioned restrictions.

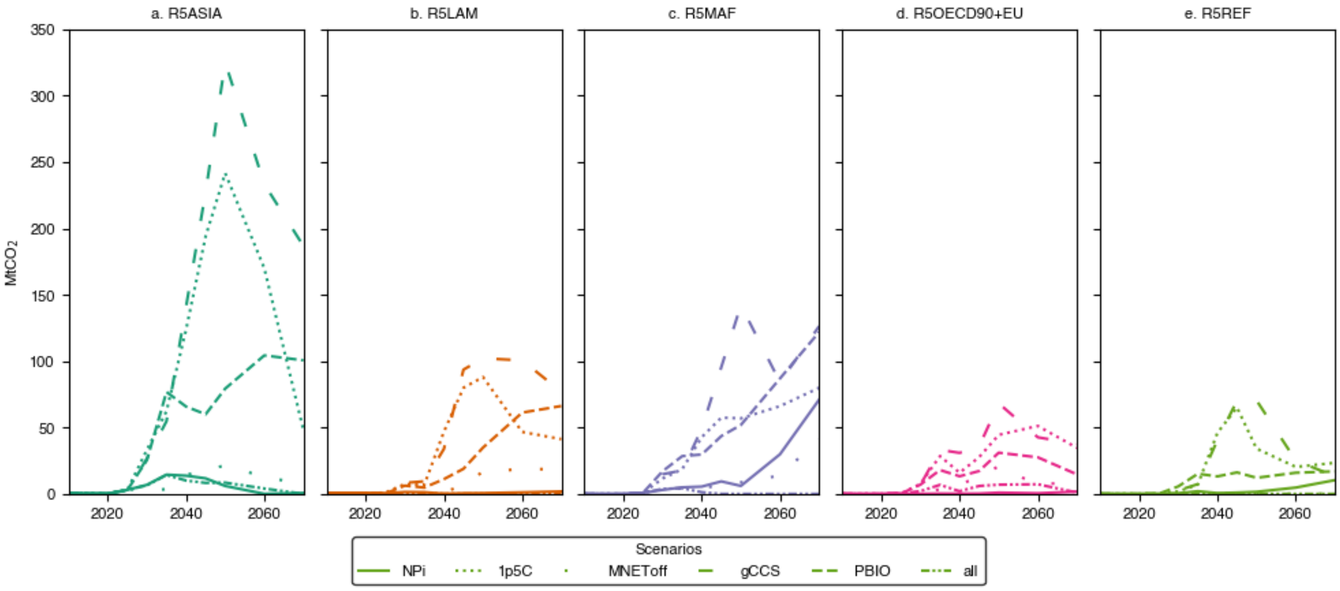

Supplementary Figure 13. Regional biogenic storage of carbon in biomaterials per R5 region. a. R5ASIA: Asia (excluding Middle East); b. R5LAM: Latin America; c. R5MAF: Middle East and Africa; d. R5OECD90+EU: OECD countries; e. R5REF: Reforming Economies (Eastern Europe, former Soviet Union). NPi: Implemented National Policies; 1.5C: carbon budget consistent with limiting global warming to 1.5°C; gCCS: a 1.5C scenario with restrictions on global CCS deployment; PBIO: a 1.5C scenario with constraints on global primary biomass use; MNEToff: a 1.5C scenario that turns off the assumption of biogenic carbon storage in materials; and all: a comprehensive 1.5C sensitivity scenario incorporating all the abovementioned restrictions.

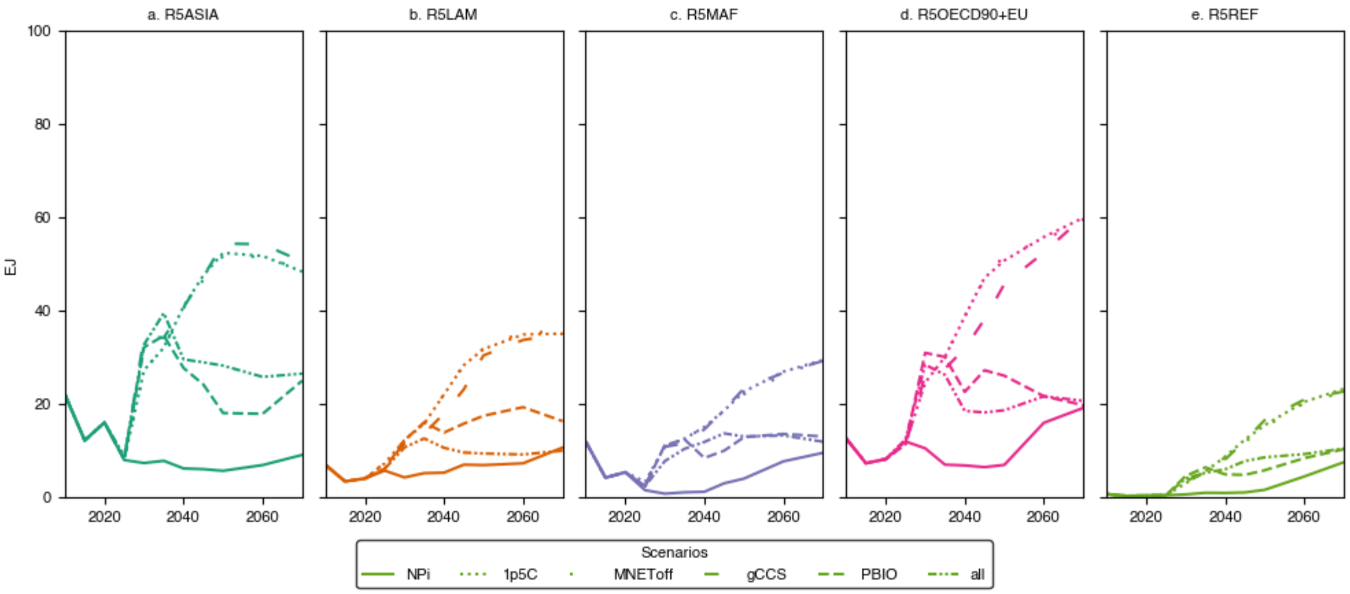

Supplementary Figure 14. Biomass use as primary energy in R5 regions. a. R5ASIA: Asia (excluding Middle East); b. R5LAM: Latin America; c. R5MAF: Middle East and Africa; d. R5OECD90+EU: OECD countries; e. R5REF: Reforming Economies (Eastern Europe, former Soviet Union). NPi: Implemented National Policies; 1.5C: carbon budget consistent with limiting global warming to 1.5°C; gCCS: a 1.5C scenario with restrictions on global CCS deployment; PBIO: a 1.5C scenario with constraints on global primary biomass use; MNEToff: a 1.5C scenario that turns off the assumption of biogenic carbon storage in materials; and all: a comprehensive 1.5C sensitivity scenario incorporating all the abovementioned restrictions. EJ: Exajoules.

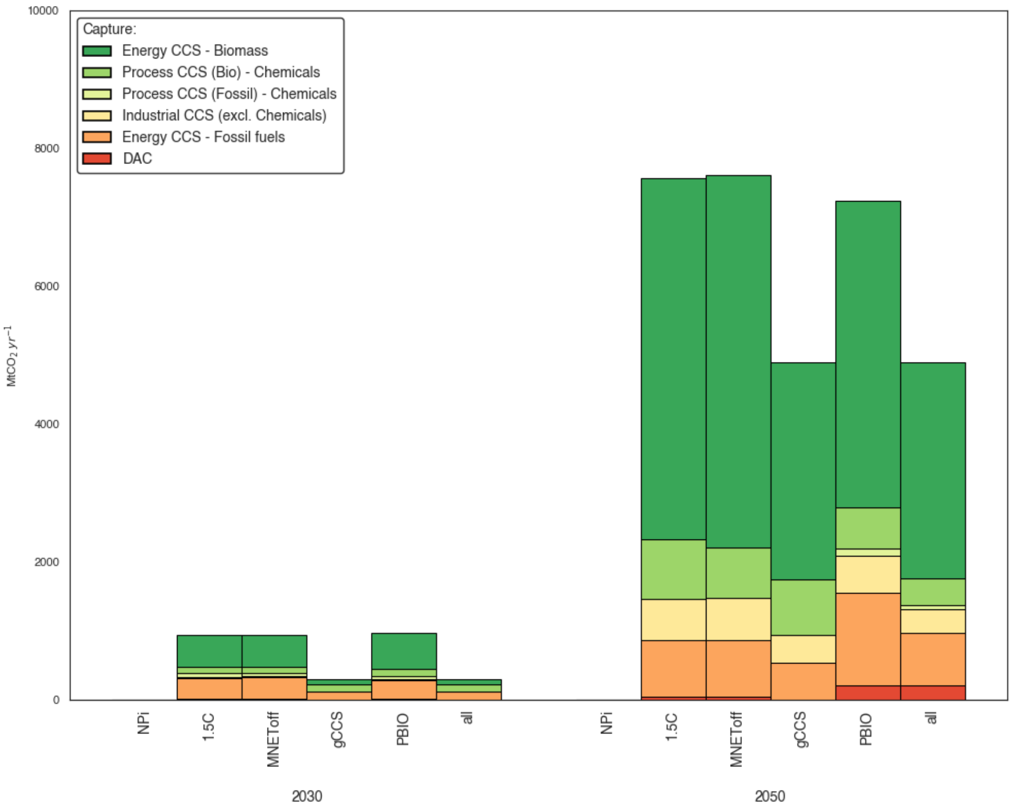

Supplementary Figure 15. Sources of carbon capture across scenarios in 2030 and 2050. NPi: Implemented National Policies; 1.5C: carbon budget consistent with limiting global warming to 1.5°C; gCCS: a 1.5C scenario with restrictions on global CCS deployment; PBIO: a 1.5C scenario with constraints on global primary biomass use; MNEToff: a 1.5C scenario that turns off the assumption of biogenic carbon storage in materials; and all: a comprehensive 1.5C sensitivity scenario incorporating all the abovementioned restrictions. DAC: Direct Air Capture. CCS: Carbon Capture and Storage.

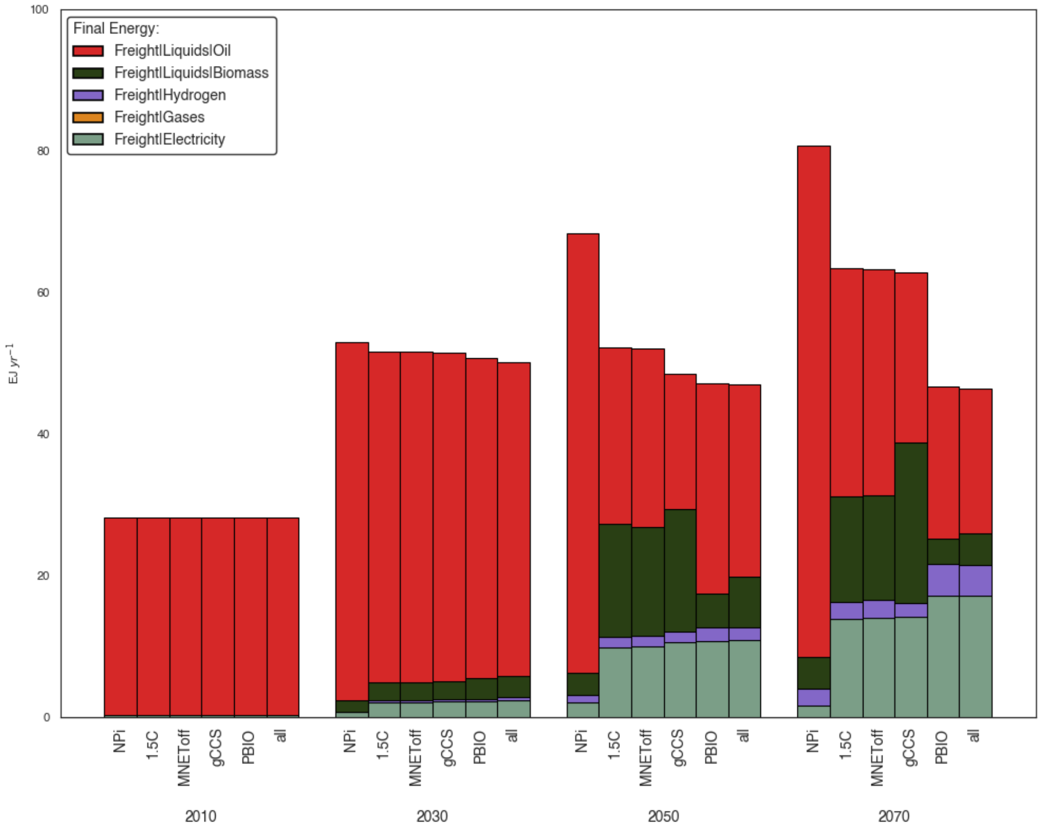

Supplementary Figure 16. Final energy use in the freight transportation sector across scenarios. NPi: Implemented National Policies; 1.5C: carbon budget consistent with limiting global warming to 1.5°C; gCCS: a 1.5C scenario with restrictions on global CCS deployment; PBIO: a 1.5C scenario with constraints on global primary biomass use; MNEToff: a 1.5C scenario that turns off the assumption of biogenic carbon storage in materials; and all: a comprehensive 1.5C sensitivity scenario incorporating all the abovementioned restrictions. EJ: Exajoules.

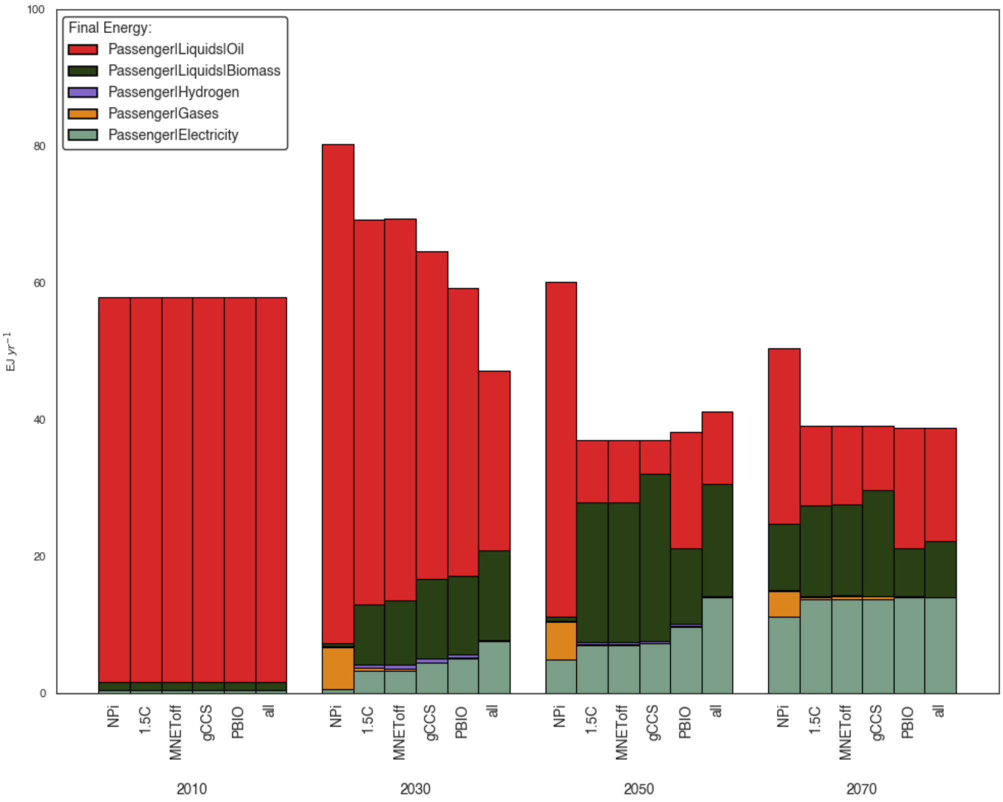

Supplementary Figure 17. Final energy use in the passenger transportation sector across scenarios. NPi: Implemented National Policies; 1.5C: carbon budget consistent with limiting global warming to 1.5°C; gCCS: a 1.5C scenario with restrictions on global CCS deployment; PBIO: a 1.5C scenario with constraints on global primary biomass use; MNEToff: a 1.5C scenario that turns off the assumption of biogenic carbon storage in materials; and all: a comprehensive 1.5C sensitivity scenario incorporating all the abovementioned restrictions. EJ: Exajoules.

Supplementary References

1. Draeger, R. *et al.* Stranded crude oil resources and just transition: Why do crude oil quality, climate ambitions and land-use emissions matter. *Energy* 124451 (2022) doi:10.1016/J.ENERGY.2022.124451.
2. Baciocchi, R., Storti, G. & Mazzotti, M. Process design and energy requirements for the capture of carbon dioxide from air. *Chem. Eng. Process. Process Intensif.* **45**, 1047–1058 (2006).
3. Socolow, R. H. *et al.* Direct Air Capture of CO2 with Chemicals: A Technology Assessment for the APS Panel on Public Affairs. in (2011).
4. IHS Markit. Global Propylene Market: Motivated to Change. http://cdn.ihs.com/www/pdf/asia-chem-conf/Carr.pdf (2014).
5. Bender, M. An Overview of Industrial Processes for the Production of Olefins – C4 Hydrocarbons. *ChemBioEng Rev.* **1**, 136–147 (2014).
6. White, W. C. Butadiene production process overview. *Chem. Biol. Interact.* **166**, 10–14 (2007).
7. Bender, M. Global Aromatics Supply - Today and Tomorrow. in *New Technologies and Alternative Feedstocks in Petrochemistry and Refining* 59–64 (2013).
8. OGJ. Worldwide Refining Survey. *Oil & Gas Journal* (2011).
9. Couch, K. A., Glavin, J. P., Wegerer, D. A. & Qafisheh, J. A. FCC propylene production. *Pet. Technol. Q.* **12**, 33–43 (2007).
10. Akah, A. & Al-Ghrami, M. Maximizing propylene production via FCC technology. *Appl. Petrochem. Res.* **5**, 377–392 (2015).
11. IHS Markit. Crude oil-to-chemicals (COTC): An industry gamechanger? (2018).
12. Ren, T., Daniëls, B., Patel, M. K. & Blok, K. Petrochemicals from oil, natural gas, coal and biomass: Production costs in 2030-2050. *Resour. Conserv. Recycl.* **53**, 653–663 (2009).
13. Ren, T., Patel, M. K. & Blok, K. Steam cracking and methane to olefins: Energy use, CO2 emissions and production costs. *Energy* **33**, 817–833 (2008).

14. OGJ. *INTERNATIONAL SURVEY OF ETHYLENE FROM STEAM CRACKERS — 2015*. 85–91 (2015).

15. Neelis, M. L., Patel, M. & Blok, K. CO2 emissions and carbon storage resulting from the non-energy use of fossil fuels in the Netherlands, NEAT results for 1993-1999. *Resour. Conserv. Recycl.* **45**, 251–274 (2005).

16. Ren, T., Patel, M. & Blok, K. Olefins from conventional and heavy feedstocks: Energy use in steam cracking and alternative processes. *Energy* **31**, 425–451 (2006).

17. Chul, Y. P., Lee, W., Young, N. & Park, D. S. Catalytic Cracking of Lower-Valued Hydrocarbons for Producing Light Olefins. 75–84 (2010) doi:10.1007/s10563-010-9089-1.

18. Eng, C. N., Kang, S. C., Choi, S., Oh, S. H. & Park, Y. K. *A Catalytic Cracking Process for Ethylene and Propylene From Paraffin Streams: The Advanced Catalytic Olefins (ACO) Process*. (2007).

19. IEA. The Future of Petrochemicals, Towards more sustainable plastics and fertilisers. (2018) doi:10.1016/B978-0-12-394381-1.00002-7.

20. Maddah, H. A. A Comparative Study between Propane Dehydrogenation (PDH) Technologies and Plants in Saudi Arabia. *Am. Sci. Res. J. Eng. Technol. Sci. ASRJETS* **45**, 49–63 (2018).

21. Monai, M. & Weckhuysen, B. M. Propane to olefins tandem catalysis: a selective route towards light olefins production. *Chem. Soc. Rev.* (2021) doi:10.1039/D1CS00357G.

22. Van Geem, K. Production of bioethylene: Alternatives for green chemicals and polymers. *AIChE Ethyl. Prod. Conf. Proc.* 303–313 (2012).

23. Tian, P., Wei, Y., Ye, M. & Liu, Z. Methanol to Olefins (MTO): From Fundamentals to Commercialization. *ACS Catal.* **5**, 1922–1938 (2015).

24. Flowserve Corporation. Coal to Olefins (CTO) / Methanol to Olefins (MTO) API Production. (2019).

25. Gogate, M. R. Methanol-to-olefins process technology: current status and future prospects. *Pet. Sci. Technol.* **37**, 559–565 (2019).

26. Xiang, D., Yang, S., Liu, X., Mai, Z. & Qian, Y. Techno-economic performance of the coal-to-olefins process with CCS. *Chem. Eng. J.* **240**, 45–54 (2014).

27. Xu, X., Liu, Y., Zhang, F., Di, W. & Zhang, Y. Clean coal technologies in China based on methanol platform. *Catal. Today* **298**, 61–68 (2017).
28. Intratec. *Propylene Production via Metathesis - Cost Analysis*. (2012).
29. Farzad, S., Mandegari, M. A. & Görgens, J. F. Integrated techno-economic and environmental analysis of butadiene production from biomass. *Bioresour. Technol.* **239**, 37–48 (2017).
30. Pomalaza, G., Arango Ponton, P., Capron, M. & Dumeignil, F. Ethanol-to-butadiene: the reaction and its catalysts. *Catal. Sci. Technol.* **10**, 4860–4911 (2020).
31. American Chemical Society. A National Historic Chemical Landmark - The Houdry Process for Catalytic Cracking. (1996).
32. Meyers, R. A. *CHAPTER 3.3: LIGHT OLEFINS/DIOLEFINS PRODUCTION VIA LUMMUS’S CATOFIN®/CATADIENE® PROCESSES*. *Handbook of Petrochemicals Production Processes, Second Edition* (McGraw-Hill Education, 2019).
33. Ullmann’s Encyclopedia of Industrial Chemistry. *Ullmanns Encycl. Ind. Chem.* (2000) doi:10.1002/14356007.
34. Guedes, F. A MULTI-REGIONAL OPTIMIZATION MODEL FOR THE BRAZILIAN OIL REFINING INDUSTRY. (Universidade Federal do Rio de Janeiro, 2019).
35. Bicer, Y., Dincer, I., Zamfirescu, C., Vezina, G. & Raso, F. Comparative life cycle assessment of various ammonia production methods. *J. Clean. Prod.* **135**, 1379–1395 (2016).
36. Dincer, I. & Bicer, Y. *Ammonia Production*. *Comprehensive Energy Systems* vols 3–5 (2018).
37. International Energy Agency. *Ammonia Technology Roadmap: Towards More Sustainable Nitrogen Fertiliser Production*. (OECD, 2021). doi:10.1787/f6daa4a0-en.
38. Smith, C., Hill, A. K. & Torrente-Murciano, L. Current and future role of Haber-Bosch ammonia in a carbon-free energy landscape. *Energy Environ. Sci.* **13**, 331–344 (2020).
39. FAO. World fertilizer trends and outlook to 2020: Summary Report. (2017).
40. FAO. Food and Agriculture Organization of the United Nations - FAOSTAT Statistical Database. (2021).

41. GPCA & Integer Research. China Fertilizer Industry Outlook to 2017 summary. (2017).
42. Dalena, F. *et al. Methanol Production and Applications: An Overview*. *Methanol: Science and Engineering* (Elsevier B.V., 2018). doi:10.1016/B978-0-444-63903-5.00001-7.
43. International Renewable Energy Agency (IRENA). *Renewable Methanol*. (2021).
44. Su, L. W., Li, X. R. & Sun, Z. Y. The consumption, production and transportation of methanol in china: A review. *Energy Policy* **63**, 130–138 (2013).
45. Zhiyan Consulting. 我国焦炉煤气制甲醇项目发展概况_智研咨询 ('The Development Situation of Coke Oven Gas to Methanol Project in China'). https://www.chyxx.com/industry/201312/225545.html.
46. Xiang, D., Yang, S., Mai, Z. & Qian, Y. Comparative study of coal, natural gas, and coke-oven gas based methanol to olefins processes in China. *Comput. Chem. Eng.* **83**, 176–185 (2015).
47. Lerede, D., Bustreo, C., Gracceva, F., Saccone, M. & Savoldi, L. Techno-economic and environmental characterization of industrial technologies for transparent bottom-up energy modeling. *Renew. Sustain. Energy Rev.* **140**, 110742 (2021).
48. SASOL b. Ammonia Information. 1–2 (2021).
49. Methanex. *Methanex Annual Reports*. https://www.annualreports.com/HostedData/AnnualReports/PDF/NASDAQ_MEOH_2022.pdf (2010).
50. IIP. Ammonia. (2015).
51. Ministry of Energy and Energy Industries | Methanol. https://www.energy.gov.tt/our-business/lng-petrochemicals/petrochemicals/methanol/.
52. EPE. Competitividade do Gás Natural: Estudo de Caso na Indústria de Metanol. *Inf. Téc.* 16 (2019).
53. Ammonia | Climate Technology Centre & Network | 1181779. https://www.ctc-n.org/technology-library/chemicals-management/ammonia.

54. Shiva Kumar, S. & Lim, H. An overview of water electrolysis technologies for green hydrogen production. *Energy Rep.* **8**, 13793–13813 (2022).
55. Carmo, M., Fritz, D. L., Mergel, J. & Stolten, D. A comprehensive review on PEM water electrolysis. *Int. J. Hydrog. Energy* **38**, 4901–4934 (2013).
56. IEA. *Extended World Energy Balances, IEA World Energy Statistics and Balances (Database)*. (2022).
57. Lantz, F., Saint-Antonin, V., Gruson, J.-F., Suwala Editor, W. & Saveyn, B. The OURSE model: Simulating the World Refining Sector to 2030. doi:10.2791/73210.
58. IEA. *The Future of Hydrogen*. (2019) doi:10.1787/1e0514c4-en.
59. Vatavuk, W. M. & others. Updating the CE plant cost index. *Chem. Eng.* **109**, 62–70 (2002).
60. Bazzanella, A. M., Ausfelder, F., & DECHEMA. Low carbon energy and feedstock for the European chemical industry. *Eur. Chem. Ind. Counc.* 168 (2017).
61. Ren, T. & Patel, M. K. Basic petrochemicals from natural gas, coal and biomass: Energy use and CO2 emissions. *Resour. Conserv. Recycl.* **53**, 513–528 (2009).
62. TechnipFMC. Ethylene Production. **225**, 1019–1022 (2017).
63. Chem, G. *et al.* Catalytic Cracking of Naphtha into Light Olefins. **240**, 2006 (2006).
64. Nesterenko, N., Aguilhon, J., Bodart, P., Minoux, D. & Dath, J. P. *Methanol to Olefins: An Insight Into Reaction Pathways and Products Formation*. *Zeolites and Zeolite-like Materials* (2016). doi:10.1016/B978-0-444-63506-8.00006-9.
65. Xu, Z., Zhang, Y., Fang, C., Yu, Y. & Ma, T. Analysis of China's olefin industry with a system optimization model – With different scenarios of dynamic oil and coal prices. *Energy Policy* **135**, 111004 (2019).
66. Venner, R. M. & Kantorowicz, S. I. Metathesis: Refinery and ethylene plant applications. *Pet. Technol. Q.* **6**, 141-142,144 (2001).

67. Salkuyeh, Y. K., Saville, B. A. & MacLean, H. L. Techno-economic analysis and life cycle assessment of hydrogen production from different biomass gasification processes. *Int. J. Hydrog. Energy* **43**, 9514–9528 (2018).
68. Müller-Casseres, E. *et al.* Are there synergies in the decarbonization of aviation and shipping? An integrated perspective for the case of Brazil. *iScience* **25**, 105248 (2022).
69. Summers, W. Baseline Analysis of Crude Methanol Production from Coal and Natural Gas. *Natl. Energy Technol. Lab.* DOE/NETL-341/101514. 1-83 (2014).
70. EUROMAP. Plastics Resin Production and Consumption in 63 Countries Worldwide 2009-2020. 80 (2016).
71. O’Neill, B. C. *et al.* The roads ahead: Narratives for shared socioeconomic pathways describing world futures in the 21st century. *Glob. Environ. Change* **42**, 169–180 (2017).
72. Stegmann, P., Daioglou, V., Londo, M., van Vuuren, D. P. & Junginger, M. Plastic futures and their CO2 emissions. *Nature* **612**, 272–276 (2022).
73. Geyer, R., Jambeck, J. R. & Law, K. L. Production, use, and fate of all plastics ever made. *Sci. Adv.* **3**, 25–29 (2017).
74. Levi, P. G. & Cullen, J. M. Mapping Global Flows of Chemicals: From Fossil Fuel Feedstocks to Chemical Products. *Environ. Sci. Technol.* **52**, 1725–1734 (2018).
75. Levi, P. G. & Cullen, J. M. Mapping Global Flows of Chemicals: Supporting Information. *Environ. Sci. Technol.* (2018).
76. Chatterton, C. *METHANOL AS AN ALTERNATIVE FUEL*. (2018).
77. OECD & FAO. *OECD-FAO Agricultural Outlook 2021-2030*. (2021).
78. Garaffa, R. *et al.* Distributional effects of carbon pricing in Brazil under the Paris Agreement. *Energy Econ.* **101**, 105396 (2021).
79. Cunha, B. S. L., Garaffa, R. & Gurgel, Â. C. TEA Model Documentation. (2020).

80. de Oliveira, C. C. N., Zotin, M. Z., Rochedo, P. R. R. & Szklo, A. Achieving negative emissions in plastics life cycles through the conversion of biomass feedstock. *Biofuels Bioprod. Biorefining* **15**, (2021).
81. World Bank Group. *WHAT A WASTE 2.0 - A Global Snapshot of Solid Waste Management to 2050*. (Washington, 2018).
82. Köberle, A. C. *et al. Can Global Models Provide Insights into Regional Mitigation Strategies? A Diagnostic Model Comparison Study of Bioenergy in Brazil*. *Climatic Change* vol. 170 (Springer Netherlands, 2022).
83. Rochedo, P. Development of a global integrated energy model to evaluate the Brazilian role in climate change mitigation scenarios. (COPPE/UFRJ, 2016).
84. Hasegawa, T. *et al.* Land-based implications of early climate actions without global net-negative emissions. *Nat. Sustain.* **4**, 1052–1059 (2021).
85. IAMC. IAMC Model Documentation. (2022).
86. European Commission. Joint Research Centre. *CO2 Emissions of All World Countries :JRC/IEA/PBL 2022 Report.* (Publications Office, LU, 2022).
87. Intergovernmental Panel On Climate Change. *Climate Change 2021 – The Physical Science Basis: Working Group I Contribution to the Sixth Assessment Report of the Intergovernmental Panel on Climate Change*. (Cambridge University Press, 2021). doi:10.1017/9781009157896.
88. Cavalett, O., Norem Slettmo, S. & Cherubini, F. Energy and Environmental Aspects of Using Eucalyptus from Brazil for Energy and Transportation Services in Europe. *Sustainability* **10**, 4068 (2018).
